# From natural wood to high-performance composites: role of fiber treatment and graphene nanoplatelets

**DOI:** 10.1038/s41598-026-64844-7

**Published:** 2026-08-08

**Authors:** M. Megahed, Aliaa Ashraf, A. A. M. Badawy

**Affiliations:** 1 Department of Mechanical Design and Production Engineering, Faculty of Engineering, Zagazig University, P.O. Box 44519, Zagazig, Egypt; 2 Materials Engineering Department, Faculty of Engineering, Zagazig University, Zagazig, 44519 Egypt

**Keywords:** Wood/epoxy composites, Alkaline treatment, Mechanical characterization, Graphene nanoplatelets, Swedish and pitch pine woods, Engineering, Materials science, Nanoscience and technology

## Abstract

Sustainable wood-epoxy composites were developed through wood fiber alkaline surface treatment and the insertion of graphene nanoplatelet (GNP). Swedish and pitch pine wood short fibers were the two types of wood used to strengthen the epoxy matrix. To enhance interfacial bonding, wood fibers were treated with two content of NaOH 5% and 10%. The epoxy matrix was supplemented with GNPs at weight percentages of 0.125 and 0.25. The results showed that composites reinforced with 5% alkali-treated wood fibers exhibited improved tensile, flexural and impact properties. However, increasing the treatment concentration to 10% led to a decrease in mechanical properties due to excessive fiber degradation. Also, the addition of GNPs gradually improved the mechanical properties; 0.125 wt% GNPs performed better than 0.25 wt% in terms of tensile, flexural and impact performance. These results show that the sustainable wood-epoxy composites with 0.125% GNPs have great potential for lightweight and eco-friendly applications, such as furniture, building panels, automobile interior parts, and other structural and semi-structural wood-based products that need improved mechanical performance.

## Introduction

Polymers have taken the place of many traditional metals and materials in a variety of applications during the past several years. This has been rendered possible by the benefits polymers provide over traditional materials. The ease of processing, productivity, and cost reduction are the main benefits of using polymers. In the majority of these applications, fillers and fibers are used for modifying the properties of polymers in order to satisfy the high strength/high modulus requirements. Since natural fibers offer better properties compared to conventional reinforcement materials, it has recently drawn the attention of scientists and technologists as the development of natural fiber composites has been a topic of interest for the past few years^1–4^. Some of the key features of the natural fibers that demonstrate their versatility as filler material in the polymer composites include decreased tool wear, decreased density per unit volume, low cost and improved specific strength, renewability, and decomposability characteristics. There are many different kinds and sizes of natural fibers that are utilized as reinforcement or fillers. Natural fiber composites produce fewer significant environmental and health risks during the manufacturing process than synthetic fiber reinforced composites like carbon and glass fibers^5,6^.

The primary issue with using natural fiber composites is that natural fibers absorb a lot of moisture, which makes the fibers and matrix less compatible^7^. While natural fibers are primarily impacted by moisture absorption, certain resins have a high moisture absorption capacity^8^. In order to improve the strength and stiffness of the natural fiber composite, it is necessary to modify both the fiber surface and the matrix to increase the adherence of natural fibers to the matrix. Through chemical reactions, chemical modification of natural fibers enhances their adherence to the matrix. To fully understand the impact of chemical treatment on natural fibers, numerous studies have been conducted. Weak bonding occurs at the interfaces of natural fiber composites due to the hydrophilic nature of natural fibers and the hydrophobic nature of matrices, which are regarded as two separate phases. When natural fibers are chemically treated, their natural hydrophilic behavior is reduced and the matrix and fiber’s adhesion qualities are enhanced^6,7^. One of the simplest, most affordable, and most effective ways to enhance the adhesion qualities of natural fibers to the matrix is the alkaline treatment approach. This technique uses sodium hydroxide (NaOH) to change the cellulosic molecular structure of natural fibers^9^. Fiber fragmentation and disaggregation develop more quickly after alkaline treatment. Alkaline treatment affects fibers in the following ways: elimination of specific quantities of oil, lignin, wax, and other contaminants; cellulose breakdown that exposes short-length crystallites. The wettability of fiber surfaces is enhanced by an increase in surface roughness, which results in improved mechanical properties^10^. Additionally, it improves bonding reaction and mechanical interlocking, which benefits fiber matrix adhesion^11,12^. Fibers are briefly treated with 5–6% sodium hydroxide (NaOH) to improve the interfacial adhesion between them and the polymer matrix. It will make the skin rougher, remove lignin, hemicellulose, wax, and oils, and lessen the amount of cellulose that is visible on the fiber surface^13^.

Wood is a naturally occurring composite of a lignin matrix and strong cellulose fiber. Over the past ten years, wood-polymer composites have become increasingly important as a result of technological advancements in materials, machinery, and processing conditions. Wood composites are used in many different building products^14^. Due to a shortage of high-quality hardwoods, researchers are driven and encouraged to search for substitute resources, such as softwood and some low-density hardwoods, for value-added applications. In order to achieve this goal, the right technologies are needed to improve the wood’s mechanical, thermal, durability, decay resistance, and hardness qualities as well as to meet certain end-use requirements^15^. If handled improperly, the wood materials’ biodegradability and high moisture absorption have detrimental effects. The chemical assembly and certain functional aspects of wood could be responsible for a number of shortcomings^16^. The primary disadvantage and constraint of wood is its deterioration due to environmental variations that affect its mechanical and physical characteristics^17,18^. Wood has many reactive sites in terms of chemistry. The hydroxyl groups, which are widely present in the three main chemical components of wood, cellulose, hemicellulose, and lignin are the most prevalent responsive sites. Wood’s physical characteristics change as a result of these OH groups absorbing humidity from humid environments. Wood experiences alternating swelling and physical deterioration due to dynamic humidity conditions. Because of its hydrogen bonding, moisture absorption readily alters the mechanical characteristics of wood^19^. In contrast, wood is an amorphous substance. The structure of the cell walls causes the porosity of both cell voids and micropores. Cell cavities are the primary routes through which moisture moves through wood^20^. Thermosetting and thermoplastic polymer systems have been introduced to reinforce the solid wood^21^. It is clear that creating wood polymer composites is an effective method to enhance the qualities of wood. Impregnation method or technique is the most collective and cheap technique to prepare wood polymer composites with a polymeric monomer or prepolymer. This method makes it simple to use polymers or monomers to fill the wood’s empty lumens. On the other hand, sodium hydroxides (NaOH) are widely used to modify the interface between different materials, like raw fibers and thermosetting or thermoplastic polymers^22^. Wood ash can be used to create composites, adding significant value. This will significantly lessen the amount of waste in our surroundings^23^.

Wood modification using nanotechnology with silicate and nanoclay reinforcement has been studied in recent years. The properties of wood were successfully improved by applying nanotechnology modification^24^. The physical, mechanical, thermal, and fracture properties of epoxy composites reinforced with micro wood particles and low concentration of graphene nanoplatelets (GNPs) were studied^25^.With varying weight percentages of wood particles (0, 2.5, 5, 7.5, and 10) and a constant 0.5% of GNPs, the hybrid composites were created using hand layup techniques. In comparison to unreinforced wood specimens, specimens with 5 wt% wood particles composites showed increases in tensile strength of 35.55%, flexural strength of 30.64%, hardness of 22.98%, impact strength of 41.67%, conductivity of 16.05%, fracture toughness of 26.71%, and fracture energy of 74.38%, respectively. The morphological analysis verified that increased wood particle loading (7.5–10%) led to agglomeration, weak particle bonding with the epoxy matrix, and consequent property reductions.

Natural composite materials, like Swedish and pitch pine woods, are gaining traction as eco-friendly alternatives to synthetics due to their renewability and high strength-to-weight ratio. While wood is a classic structural material, repurposing pine wood waste into polymer-based composites offers an understudied opportunity to advance the circular economy. By converting industrial waste into high-value engineering materials, this approach reduces environmental impact while providing durable, cost-effective solutions for the future.

Integrating graphene nanoplatelets (GNPs) into natural composites, like Swedish and pitch pine woods, addresses issues such as weak interfacial bonding and mechanical inconsistency. GNPs improve stress transfer and prevent crack propagation, leading to a more homogeneous microstructure with enhanced tensile and flexural strength. The incorporation transforms sustainable wood composites into high-performance materials suitable for advanced structural applications.

This research aims to fabricate wood-epoxy composites utilizing Swedish wood and pitch pine wood as natural fibers. To enhance the interfacial bonding and overall performance, the wood reinforcements were subjected to alkaline treatments at concentrations of 5% and 10% NaOH. Furthermore, graphene nanoplatelets were incorporated into the epoxy matrix to enhance the mechanical properties of the resulting composites with different weight percentages. Tensile, flexural and impact tests were performed on all types of fabricated natural composites.

## Experimental work

### Materials

Pitch pine wood (PW) and Swedish wood (SW) were the two types of wood fibers utilized as natural reinforcement for epoxy. These two types of wood were provided by united company for wood, Egypt. Prior to the creation of wood-fiber epoxy composites, different chemical treatment ratios were applied to each type of wood reinforcing. NaOH was used in ratios of 5% and 10% for alkali treatment. Kemapoxy 150 RGL and amine/polyamine hardener was the epoxy system and was provided by CNB Company. The graphene nanoplatelet was supplied by Aldrich Company. The GNP size was 5 µm, 6–8 nm thickness and a surface area of 120–150 m^2^/g. Sodium hydroxide used in the alkali treatment of wood fibers were supplied by El Nasr Pharmaceutical Chemicals, Egypt. The natural composite specimen’s designation was displayed in Table 1. Tensile, flexural and impact test on treated and untreated wood fiber epoxy composites with and without nanofillers were performed.

**Table 1:** The designation of the natural composite specimen.

Designation	Type of wood fiber	NaOH %	GNPs wt%
P0	PW	0	0
PT5	PW	5	0
PT10	PW	10	0
S0	SW	0	0
ST5	SW	5	0
ST10	SW	10	0
N1	SW	0	0.125
N2	SW	0	0.25

### Manufacturing of wood epoxy composites

#### Chemical treatment of pine and Swedish wood short fibers

Alkali treatment is a popular chemical modification technique for natural fibers, particularly wood fibers, that increases the fibers’ compatibility with polymer matrices such as epoxy resin. This method involves applying an aqueous solution of sodium hydroxide (NaOH) to the fibers. In this investigation, wood/epoxy samples were kept in aqueous solutions containing either 5% or 10% sodium hydroxide (NaOH) at room temperature for a whole day. Following this initial soaking time, the liquids and the soaked wood were boiled for two hours. After that, the wood was cleaned of any NaOH leftover. After that, the wood was allowed to cool naturally in the sun for seven days. The chemically altered wood was ultimately dried using a natural evaporation procedure to remove any last traces of moisture.

#### Manufacturing of wood/epoxy composites

A set weight fraction of 10% was used to introduce wood fibers to the matrix. Wood fibers, both treated and untreated, were manually mixed until they were dispersed uniformly throughout the epoxy matrix. This method needed the continuous application of mechanical energy through stirring in order to overcome the attractive forces between the fibers and enable the disentanglement and even dispersion of individual fibers inside the epoxy matrix. Any agglomeration or clumping had to be removed in order to ensure that the fibers were evenly distributed and individually suspended throughout the volume. This procedure is crucial for achieving consistent material properties in further processing or analysis.

Once the wood fibers were properly and uniformly distributed throughout the polymeric epoxy matrix, the finished material was poured into a glass mold that had been previously waxed. A 2-cm-deep mold with exact dimensions of 40 cm in length and 30 cm in width was utilized. The well-dispersed wood fibers enhance the mechanical qualities of the composite, such as its tensile and flexural strength, while the glass mold’s flat surface and transparency allow for the observation of the curing process.

#### Fabrication of natural nanocomposite

After the wood composite samples were made and their performance evaluated, the best-performing sample was selected for further modification. To investigate the effects of adding nanofiller on the properties of the composite, graphene nanoplatelets were added to this sample at two distinct weight fractions: 0.125 and 0.25% from epoxy’s weight. Before adding epoxy, the nano graphene was also weighed, calculated, and thoroughly mixed using a glass rod stirrer. After that, this combination was sonicated for two hours. A Hielscher ultrasonic processor UP 200S with a 0.5 s on/off cycle was utilized for mixing at an amplitude of 40%^26^. During the sonication process, the mixture was immersed in an ice and water solution to lessen the heat generated by the operation^27^. Sonication is the process of employing sound waves to disperse nano particles in an epoxy solution. These interruptions can be employed to speed up the dissolution of a solid into the epoxy, break up the agglomeration of nano particles, and eliminate dissolved gas from the mixture. The liquid was then thoroughly mixed and a hardener was added at a 2:1 ratio. A magnetic stirrer was used to mix the hardener for 10 min at 250 rpm in order to promote the dispersion of nanoparticles and break up agglomeration, which improves the compatibility of nanopaticles with epoxy resin.

**Fig. 1:**
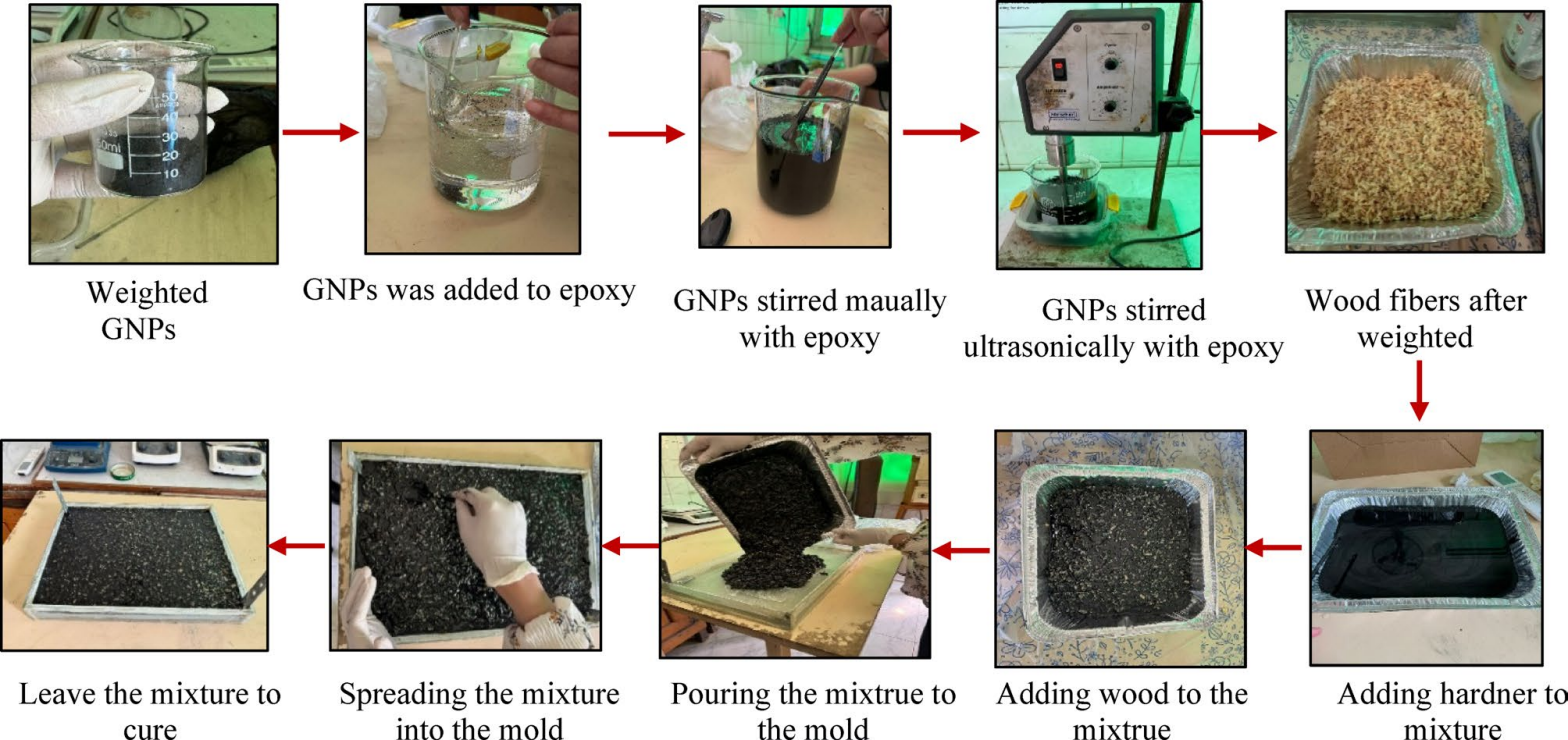
Nano/Natural composite fabrication.

Following the incorporation of graphene nanoparticles into the epoxy resin, wood fibers were gradually added to the modified epoxy matrix. The fibers were then evenly disseminated throughout the matrix by physically stirring the liquid. After homogeneity was ensured, the finished composite mixture was poured into a glass mold that had previously been lightly coated with wax to avoid adherence. The dimensions of the mold were 40 by 30 by 2 cm. In order to get the best matrix-fiber bonding and complete solidification, the composite was then left to cure 7 days in the natural environment. Figure 1 shows the steps of nanocomposite manufacturing.

### Experimental tests

The Ibertest universal testing machine (testcom-100) was used to perform tests for tensile and flexural tests. The testing machine’s computer unit provided the load deflection curves.

#### Tensile test

**Fig. 2:**
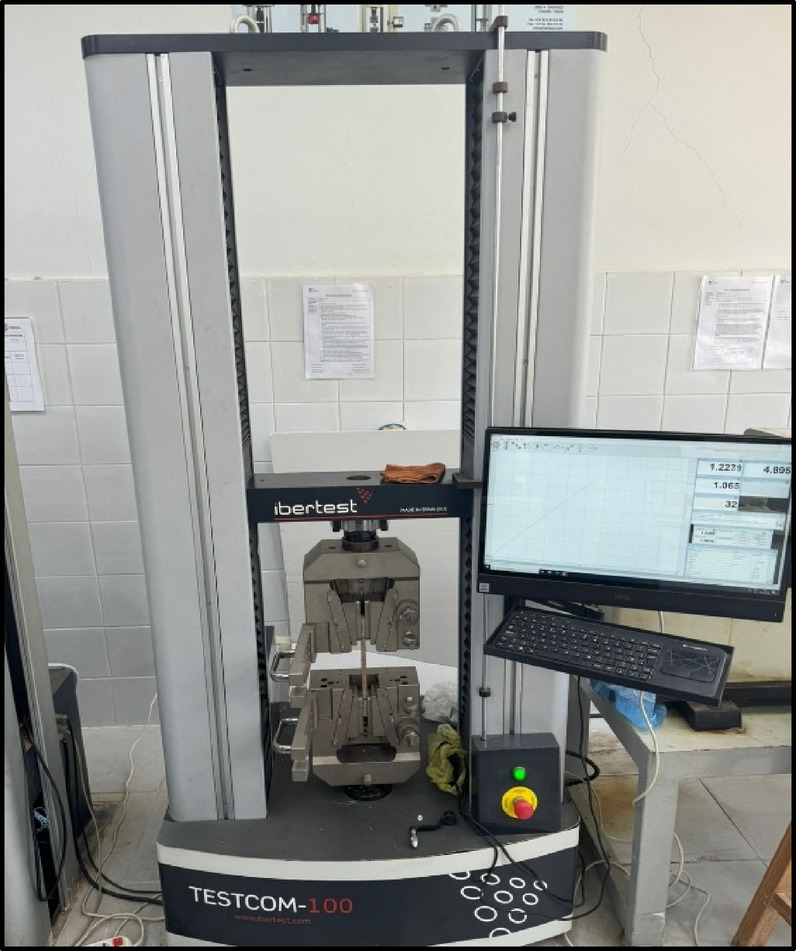
Ibertest universal testing machine (testcom-100).

ASTM D3039/D3039M specifications were used to estimate the tensile testing properties (young’s modulus, strain to failure, and ultimate tensile strength)^28^. The test was conducted using a computerized universal testing apparatus (Ibertest universal testing machine (testcom-100)) as shown in Fig. 2. The cross-head speed that was utilized was 2 mm/min. The test specimen was cut from the composite sheet and measured 250 mm in length and 25 mm in width. Five specimens were tested for each type of natural composites. A computer collecting data system automatically recorded the stress–strain curves. Young’s modulus (${E}_{app}$), strain to failure (${\varepsilon}_{l}$), and ultimate tensile strength (${\sigma}_{ult}$) were all simply estimated.

#### Flexural test

**Fig. 3:**
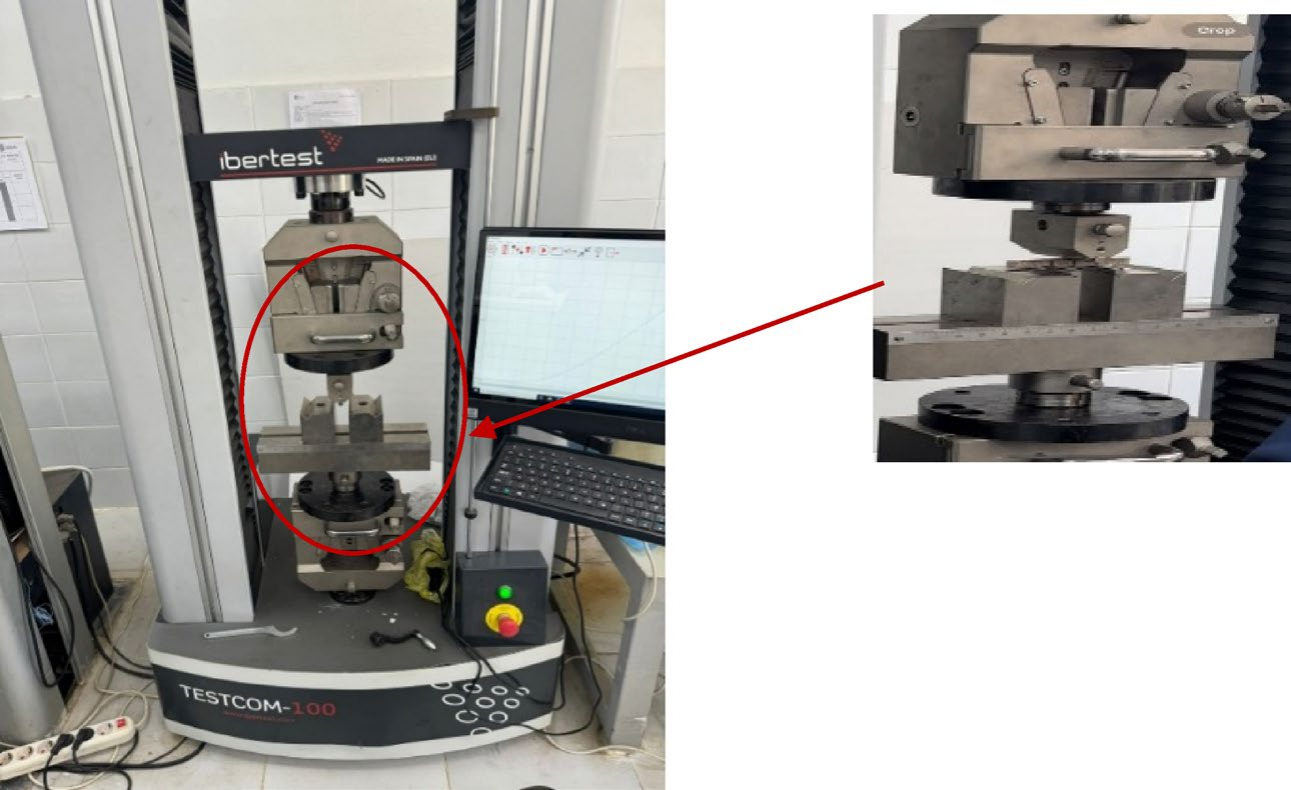
Flexural testing machine set-up.

In accordance with ASTM D790–10, three-point bending tests have been performed at a steady cross-head rate of 2 mm/min^29^. The dimensions are 150 mm in length, 60 mm in span, and 15 mm in width. The test was conducted using a computerized universal testing apparatus (Ibertest universal testing machine (testcom-100)) as shown in Fig. 3. The bending test jig was fastened to the universal testing machine grips. Five specimens were tested for each type of natural composites. The following formula was used to determine the flexural strength (${\sigma}_{fl}$), flexural strain (${\varepsilon}_{fl}$), and flexural modulus (${E}_{fl}$).

1 $${\sigma}_{fl}=\frac{3{P}_{max}L}{2b{h}^{2}}$$

where *f* is the applied load, *l* is the support span, and *b* and *h* are the specimen’s width and thickness, respectively. However the following formula was used to determine the modulus of elasticity in bending:

2 $${E}_{fl}=\frac{\frac{P}{\delta }{L}^{3}}{4b{h}^{3}}$$

The flexural strain was investigated from the following equation:

3 $${\varepsilon}_{fl}=\frac{6\delta h}{{L}^{2}}$$

where $\frac{P}{\delta }$ is the slope of the initial linear portion of the load–deflection curve, *L* represents the support span length, which is 60 mm, *b* is the specimen width, *h* is the specimen thickness,*δ* is the deflection and ${P}_{max}$ is the maximum load.

#### Impact test

Izod test was used according to with ASTM 256-04^30^. Specimen was subjected to a flat-wise impact test; the dimensions of the impact specimens are 46 mm length and 12.5 mm width. The machine’s pendulum has a falling velocity of 3.65 m/s and an impact energy of 5 Joule. Five specimens were tested for each type of natural composites. The impact strength was calculated by dividing the absorbed energy by the specimen’s initial cross-section area.

4 $${\mathrm{S}}_{\mathrm{i}\mathrm{m}\mathrm{p}}= \frac{\mathrm{A}\mathrm{b}\mathrm{s}\mathrm{o}\mathrm{r}\mathrm{b}\mathrm{e}\mathrm{d} \mathrm{e}\mathrm{n}\mathrm{e}\mathrm{r}\mathrm{g}\mathrm{y}}{\mathrm{S}\mathrm{p}\mathrm{e}\mathrm{c}\mathrm{i}\mathrm{m}\mathrm{e}\mathrm{n} \mathrm{c}\mathrm{r}\mathrm{o}\mathrm{s}\mathrm{s}-\mathrm{s}\mathrm{e}\mathrm{c}\mathrm{t}\mathrm{i}\mathrm{o}\mathrm{n} \mathrm{a}\mathrm{r}\mathrm{e}\mathrm{a}}$$

#### Density of the fabricated specimen

The density of the natural composites directly influences its mechanical properties and durability. To calculate the density of a wood-epoxy composite, experimental density was determined according to ASTM D792 as following^31^:

5 $$\rho =\frac{m}{V}$$

As *m* is the total dry mass of the natural composite specimen and V is the total volume of the specimen.

#### Hardness test

Barcol hardness testing is a technique used to assess the hardness of rigid plastic materials. The hardness value of the plastic material determines how resistant it is to scratches. The composite specimen is placed under the indenter of the Barcol hardness tester, and the sample is uniformly compressed until the dial indicator reaches its maximum value. The Barcol impressor hardness tester (PCE-1000N) was used to determine the hardness of the composite. For every manufactured composite sample, the hardness was measured ten times at random. The hardness value of the composite was determined by taking the average value for each type of manufactured natural composite^31^.

#### Microstructure examination

Scanning electron microscope (SEM) was used to examine the morphology of the surfaces of the wood/epoxy composites with a FEI Quanta FEG 250, USA instrument at a potential of 20 kV. All specimens for SEM examination were gold sputter coated to make the surface electrically conductive. However, Fourier Transform Infrared (FTIR) spectroscopy is used to analyze changes in the chemical functional groups of wood fibers after treatment of alkali (NaOH) treatment. The presence of functional group was characterized by ATR-FTIR Spectroscopy, Thermo Niclot, 50.

#### Thermogravimetric analysis (TGA)

Thermogravimetric analysis (TGA) was employed to evaluate the thermal stability, decomposition kinetics, and composition of the untreated and alkali-treated wood-epoxy composites. Testing was performed using a high-precision Q500 thermogravimetric analyzer (TA Instruments, USA). For each measurement, a representative sample weighing approximately 10–15 mg was placed inside a high-purity crucible.

To prevent premature oxidative degradation during the heating phases and ensure pure thermal pyrolysis, the furnace chamber was continuously purged with a steady flow of nitrogen (N_2_) gas at a controlled rate of 50 mL/min. The samples were heated from 25 °C to a maximum temperature of 600 °C at a constant, linear heating rate of 10 °C/min. Mass loss as a function of temperature was continuously monitored and recorded by the system’s microbalance.

The primary thermal parameters extracted from the resulting thermograms included the onset degradation temperature (T_onset_), the temperature at maximum degradation rate (T_max_) derived from the first derivative (DTG) curves, and the final residual char yield at 600 °C. These parameters were systematically evaluated to establish the structural correlation between chemical surface modification, fiber degradation, and the final composite performance.

## Results and discussion

### Mechanical properties of wood /epoxy composites

#### Tensile test

**Fig. 4:**
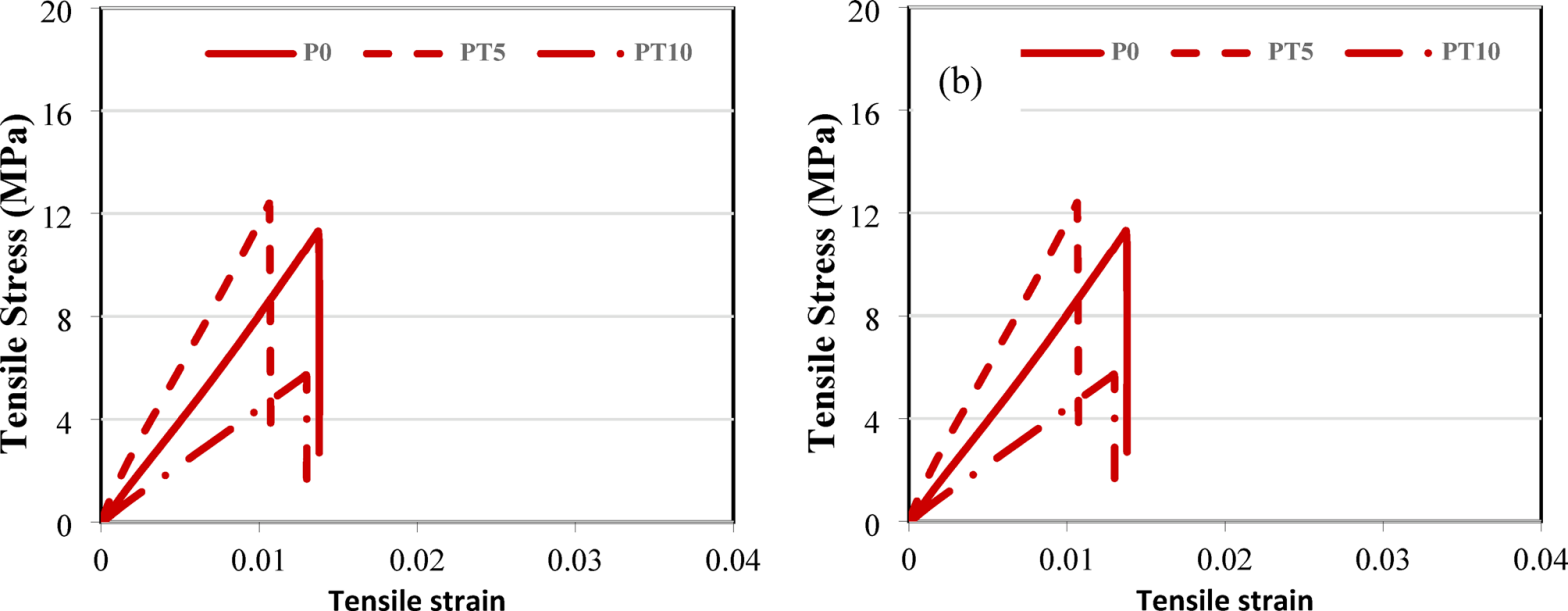
Tensile stress–strain curves of (**a**) Swedish wood/ epoxy composites, and (**b**) Pine wood/epoxy composites.

Figure 4 presents the tensile stress–strain curves of untreated and treated Swedish and Pine wood/epoxy composites. All specimens exhibits an initial nearly linear elastic region, indicating elastic deformation dominated by the epoxy matrix and effective stress transfer to the wood fibers.

Amongst of all the samples, 5% alkali treated composites (ST5 and PT5) had the highest tensile strength and a better slope in the elastic area, which suggests a higher tensile modulus. The main cause of the improvement is the 5% alkali treatment, which successfully eliminates hemicellulose and surface impurities while making the fibers’ surfaces rougher^32^. More effective load transfer from the matrix to the reinforcing fibers is made possible by this treatment, which improves fiber–matrix interfacial bonding and mechanical interlocking^33^.

**Fig. 5:**
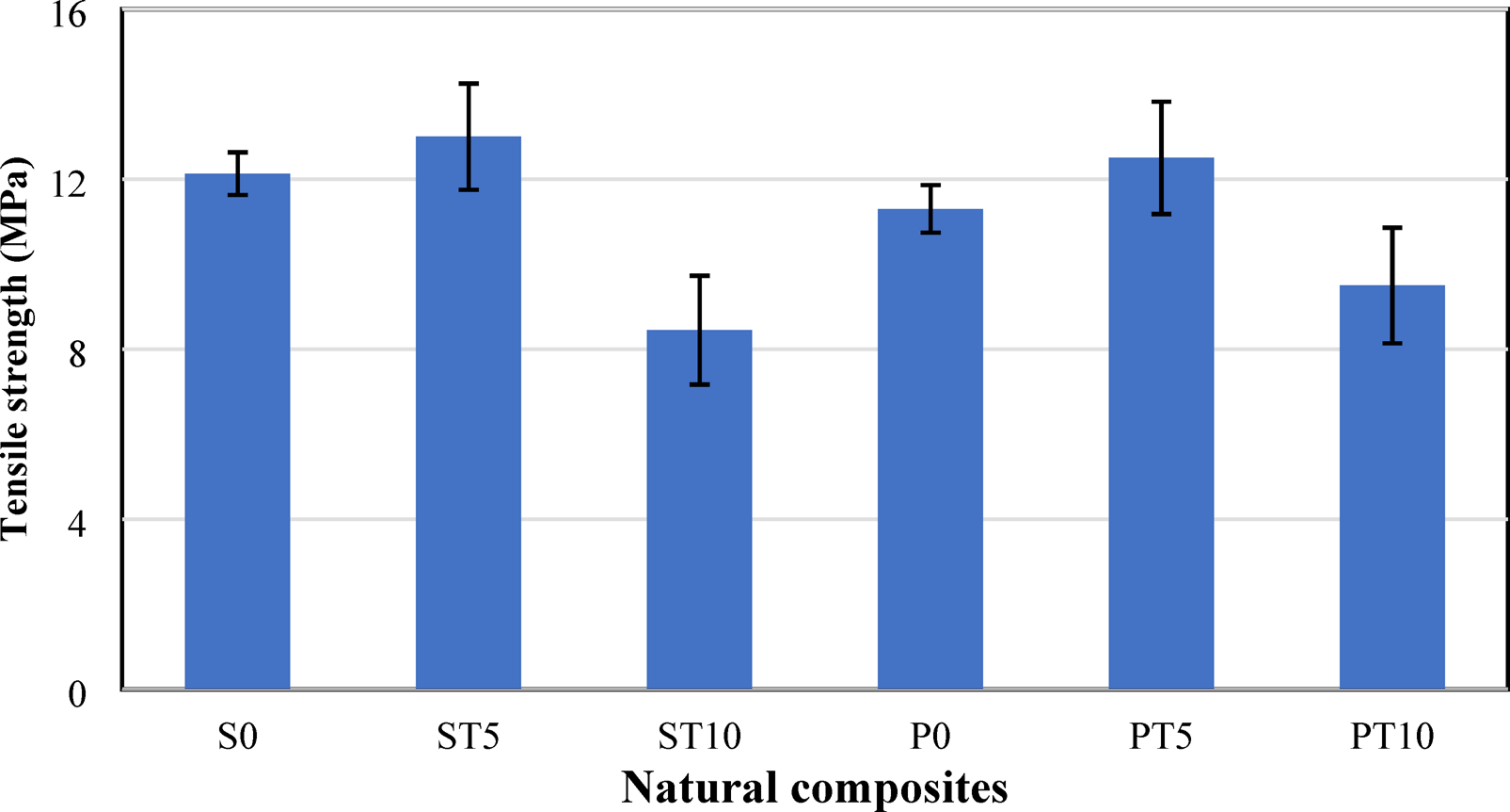
Tensile strength of treated and untreated wood/epoxy composites.

**Fig. 6:**
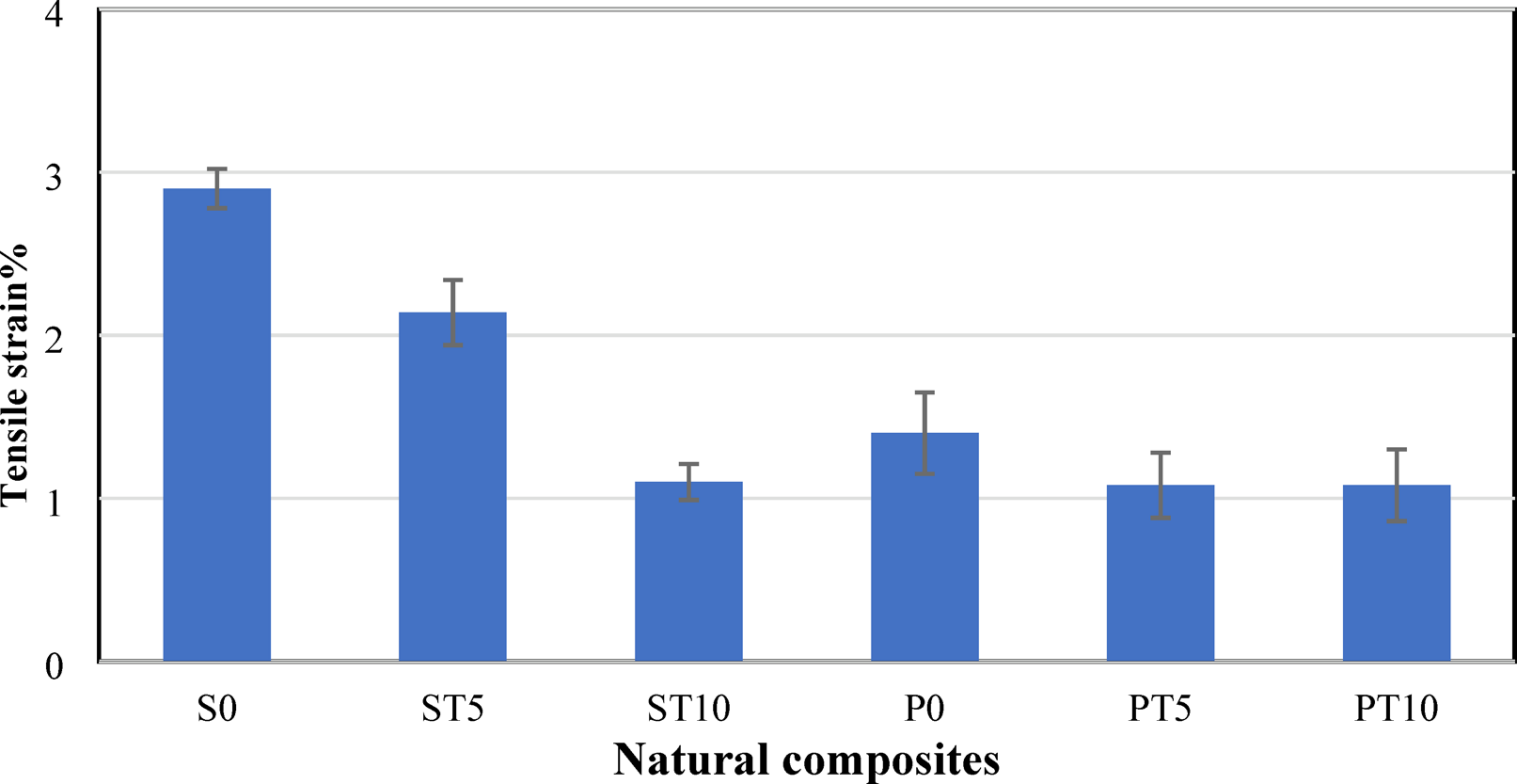
Tensile strain of treated and untreated wood/epoxy composites.

On the other hand, as compared to both untreated wood composites and 5% treated wood composites, 10% treated composites (ST10 and PT10) composites exhibited a decrease in tensile strength and strain at failure as shown in Figs. 5 and 6. Excessive alkali treatment at 10% probably causes fiber damage and partial cellulose structural collapse. The integrity of the fiber and its ability to handle loads can be damaged by excessive surface etching. Furthermore, if amorphous components are removed excessively, the fiber-matrix interface may develop microvoids and flaws that encourage early crack initiation and propagation under tensile strain^34^. Similar results were absorbed by Ismail et al.^35^.

Also, the mechanical properties of the composites decreased with the increase in the concentration of NaOH above a certain level of treatment as reported by^36,37^. This may be attributed to that when fibers are treated in too high concentration of treatment substances, inevitable damage is caused to the fibers. Besides, according to Hamidon et al*.*^38^, excessive delignification of natural fiber can result from a higher percentage of alkaline content, which weakens or destroys the fiber. Increase in the concentration of NaOH has also been observed to significantly reduce the tensile strength of the resulting composite. Even if the interface is rough, it cannot efficiently transfer loads from the matrix to the reinforcement if the alkali sufficiently damages the fibers. Because the fibers fail before the composite can reach its potential maximum load, the tensile strength decreases as a result.

A previous study showed that 5% alkali treatment is the optimum treatment level as epoxy resin was chosen as the matrix material and jute fibers as the reinforcement in this project. Using concentrations of 1%, 3%, 5%, 7%, and 9% NaOH solution, the impact of alkali treatment on different mechanical properties of jute/epoxy composites was examined. According to a number of test results, impact strength increases up to 7% concentration of NaOH before decreasing, although tensile and flexural strength increases up to 5% concentration of NaOH. By examining morphology using Fourier transform infrared analysis and scanning electron microscopy, the outcomes of mechanical characterizations were further confirmed. It has been proposed that 5% is the ideal concentration for alkali treatment of fibers^39^.

**Fig. 7:**
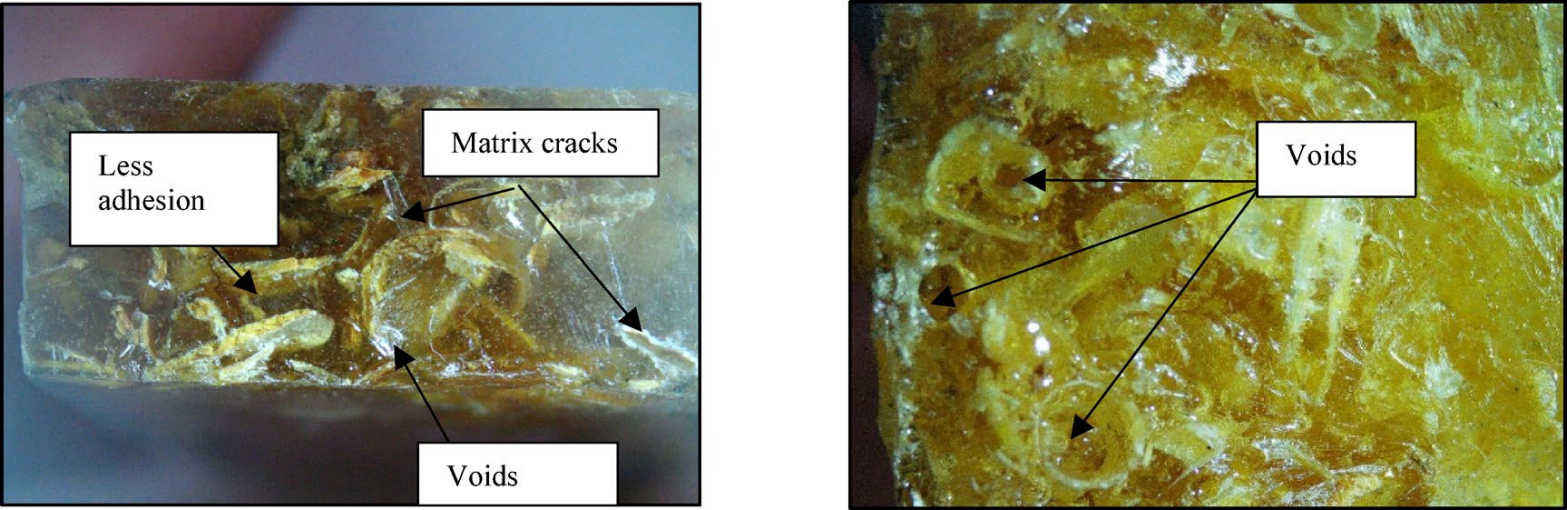
The tensile fracture surface for the untreated Swedish wood/epoxy specimens.

**Fig. 8:**
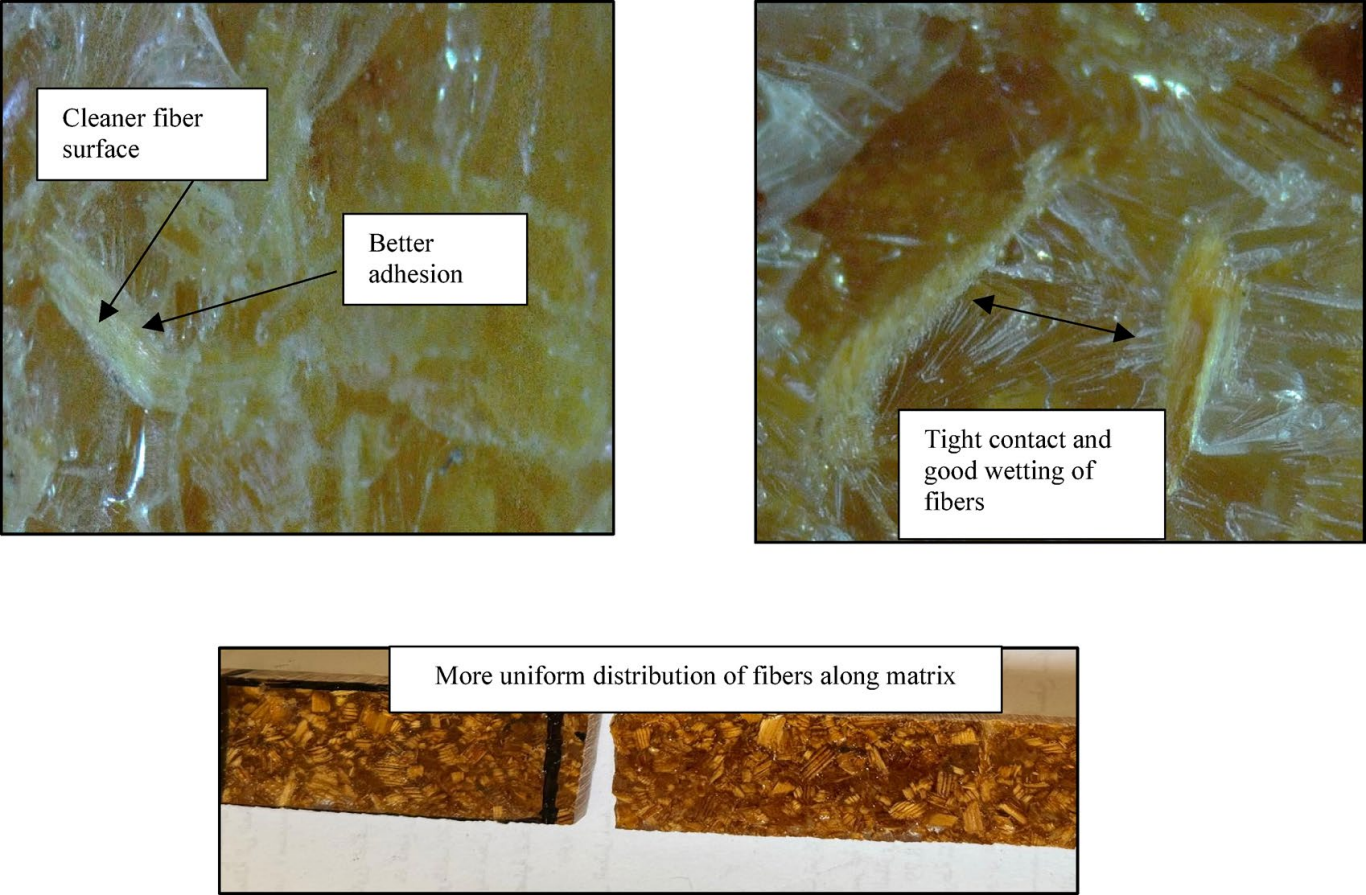
The tensile fracture surface for 5 wt% treated Swedish wood/epoxy specimens, ST5.

**Fig. 9:**
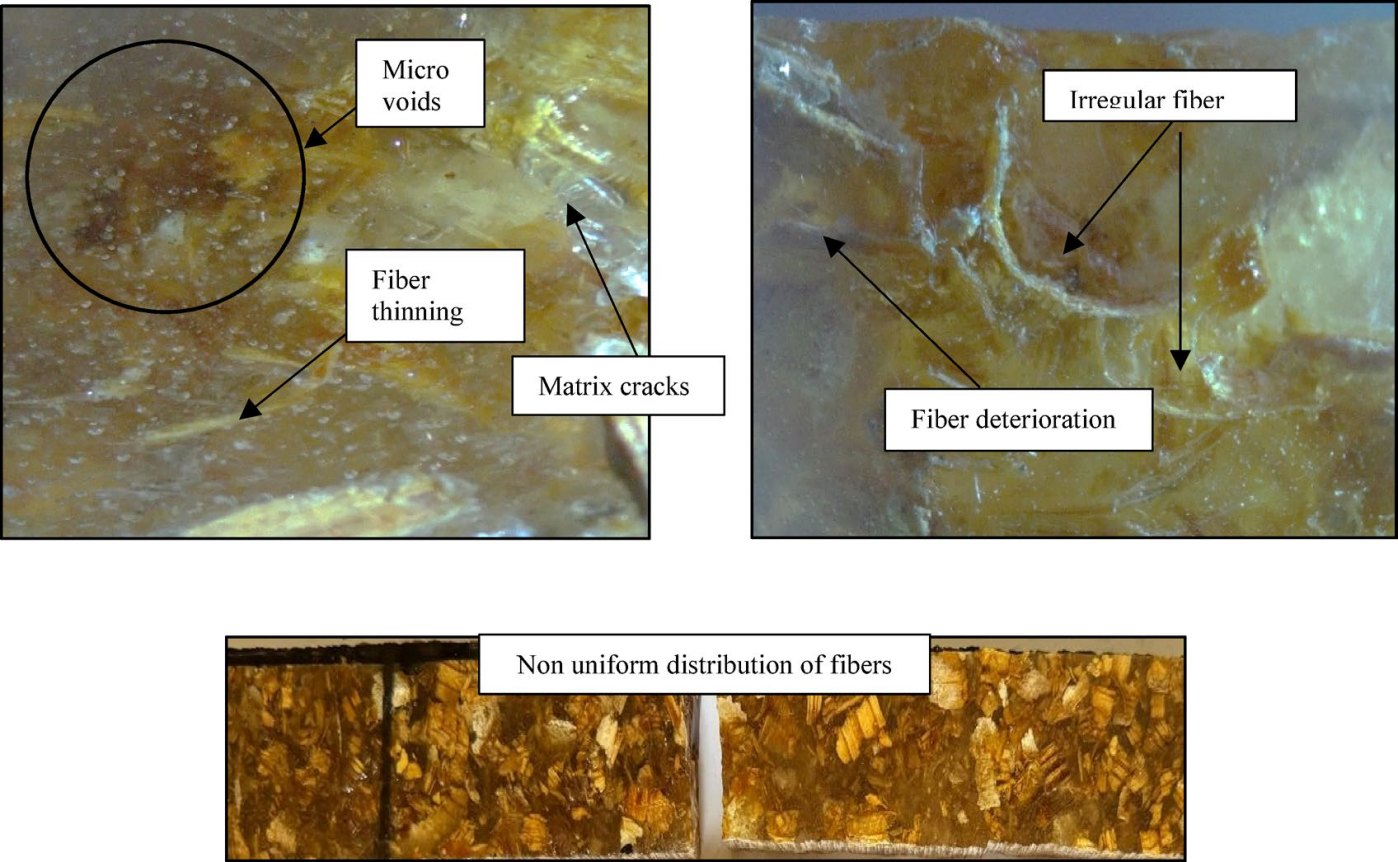
The tensile fracture surface for 10 wt% treated Swedish wood/epoxy specimens, ST10.

As shown in the Figs. 7, 8, and 9, the optical microscopy observations reveal clear differences in the microstructural features of the wood–epoxy composites as a function of alkali treatment. The untreated wood–epoxy composite exhibited noticeable voids and only moderate interfacial adhesion between the fibers and the epoxy matrix, along with a relatively non-uniform fiber distribution. These features suggest limited wetting of the fibers by the matrix, which can act as stress concentration sites and negatively affect load transfer which caused matrix cracking due to the weak load transfer. In contrast, the composite containing 5% alkali-treated wood fibers showed a more homogeneous fiber distribution within the matrix, improved fiber–matrix interfacial bonding due to tight contact between fibers and matrix, cleaner fiber surfaces, and a significant reduction in void content. This improvement can be attributed to the removal of surface impurities such as hemicellulose and waxes during moderate alkali treatment, enhancing fiber roughness and matrix penetration. However, increasing the alkali concentration to 10% resulted in a deterioration of the microstructure, characterized by increased void formation, extensive microcracking, thinning fibers, irregular fiber shapes, and non-uniform fiber dispersion. These defects indicate fiber damage and excessive delignification caused by over-treatment, which weakens the fiber structure and compromises the integrity of the fiber–matrix interface. Overall, the optical micrographs confirm that a 5% alkali treatment provides an optimal balance between surface modification and fiber integrity, while excessive treatment leads to microstructural degradation. Which was indicated and studied in our results above.

**Fig. 10:**
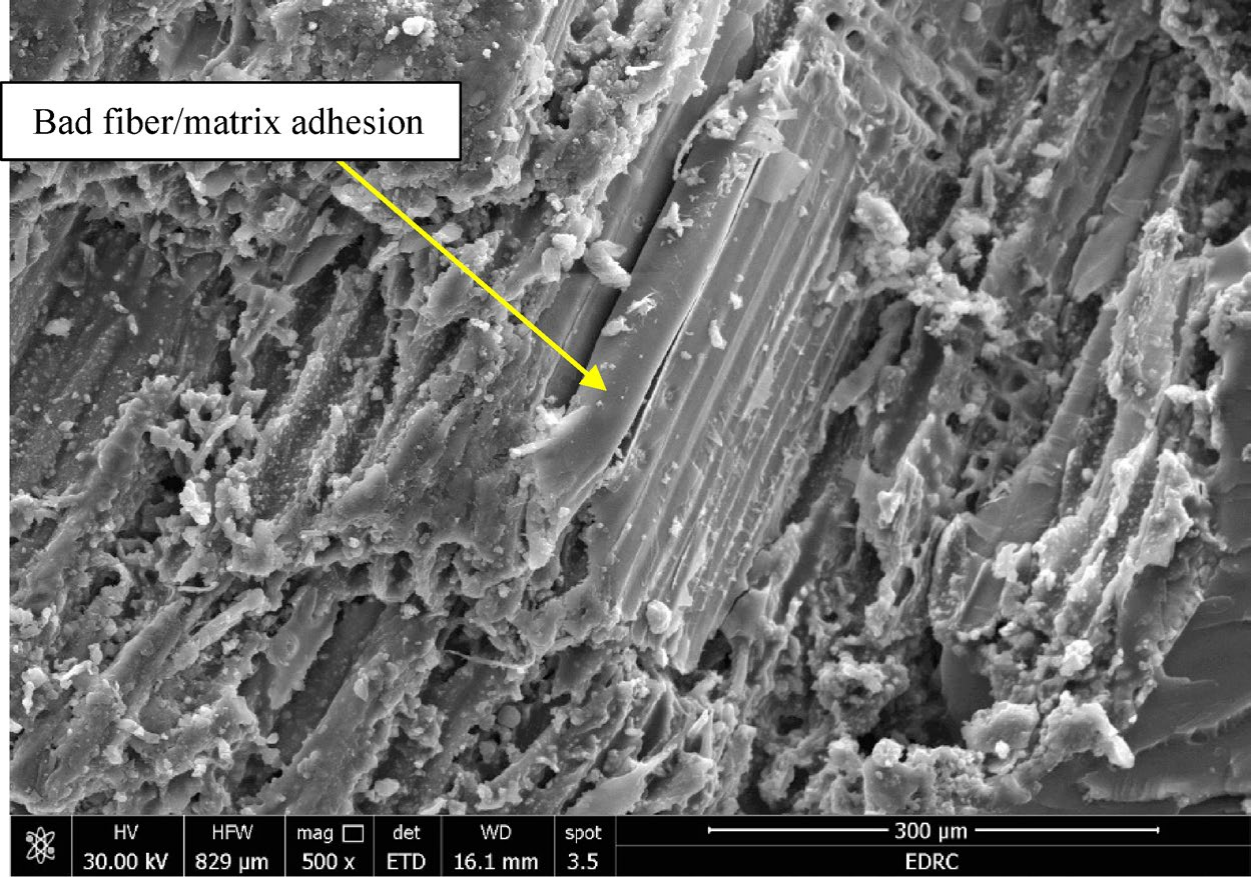
SEM images for fracture surfaces for S0 specimens after subjecting to tensile loading.

**Fig. 11:**
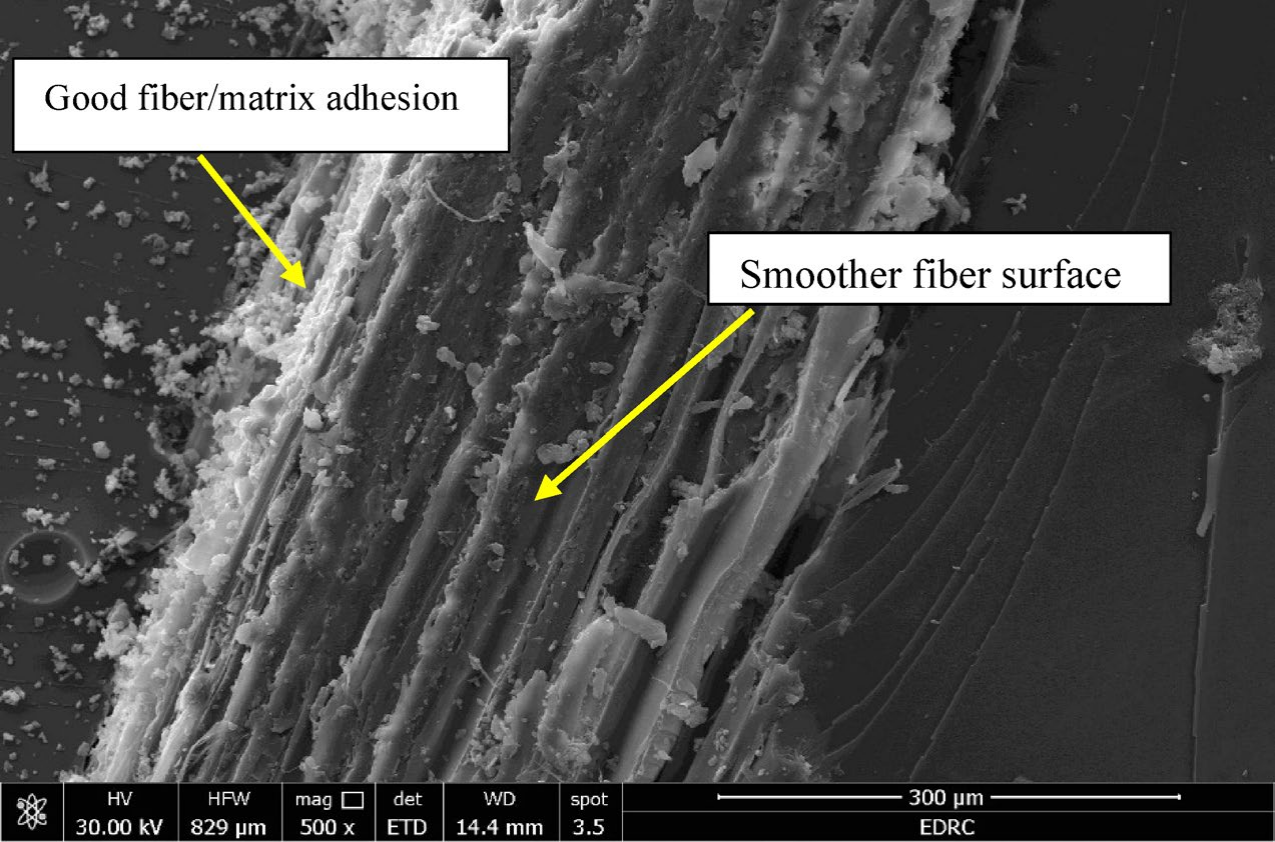
SEM images for fracture surfaces for S5 specimens after subjecting to tensile loading.

**Fig. 12:**
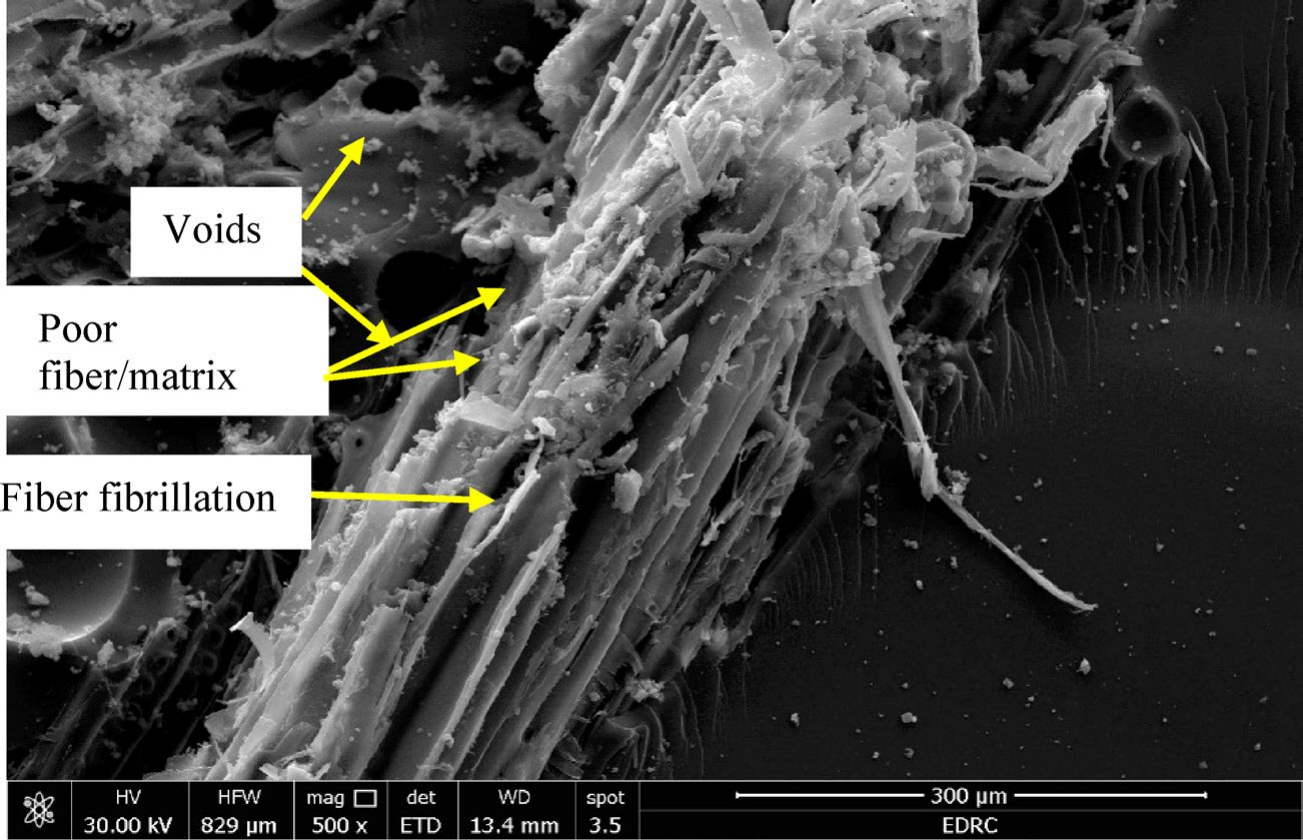
SEM images for fracture surfaces for S10 specimens after subjecting to tensile loading.

As shown in Fig. 10, the Scanning Electron Microscopy (SEM) micrographs reveal distinct variations in the interfacial morphology of the wood-epoxy composites based on the chemical treatment concentration. The untreated samples exhibit severe microstructural defects, characterized by large internal voids, prominent matrix cracking, and pronounced epoxy wrinkling. These irregularities stem directly from the poor interfacial bonding and chemical incompatibility between the hydrophilic wood fibers and the hydrophobic epoxy matrix^40^. In contrast, as shown in Fig. 11 the 5% alkali-treated composites display a highly optimized morphology. This optimal sodium hydroxide concentration effectively removes surface impurities, hemicellulose, and lignin. This chemical modification yields a smoother topography with minimal voids and a drastic reduction in matrix cracking, indicating enhanced interfacial adhesion and efficient stress transfer^41^. However, increasing the alkali concentration to 10% induces detrimental structural changes. The excessive NaOH exposure leads to severe fiber fibrillation, extensive surface damage, and the formation of micro-voids as shown in Fig. 12. This over-treatment degrades the natural cellulose structure, triggering matrix fracturing and localized debonding due to the weakened fiber core and compromised interface^42^.

As shown in Fig. 10, SEM analysis provides critical insights into the surface morphology and wetting behavior of the isolated wood fibers within the epoxy matrix. The untreated fibers exhibit poor resin impregnation, leaving distinct gaps at the interface. This lack of wettability confirms the inherent incompatibility between the hydrophilic untreated fiber surfaces and the hydrophobic epoxy resin, which severely limits mechanical interlocking. Conversely, the 5% alkali-treated fibers demonstrate a markedly smoother surface topography as shown in Fig. 11. This modification is attributed to the successful dissolution of amorphous constituents, specifically hemicellulose and surface impurities. The removal of these non-cellulosic layers increases surface roughness on a micro-scale, optimizing the fiber surface area and facilitating an excellent interfacial bond with the surrounding epoxy matrix^43^. However, escalating the chemical treatment to a 10% alkali concentration yields a highly detrimental effect as shown in Fig. 12. Excessive sodium hydroxide exposure causes severe fiber deterioration, structural degradation, and extensive fibrillation. This over-treatment strips away vital structural components of the cell wall, compromising the intrinsic strength of the fibers and leading to localized matrix debonding under mechanical stress^44^.

#### Flexural test

**Fig. 13:**
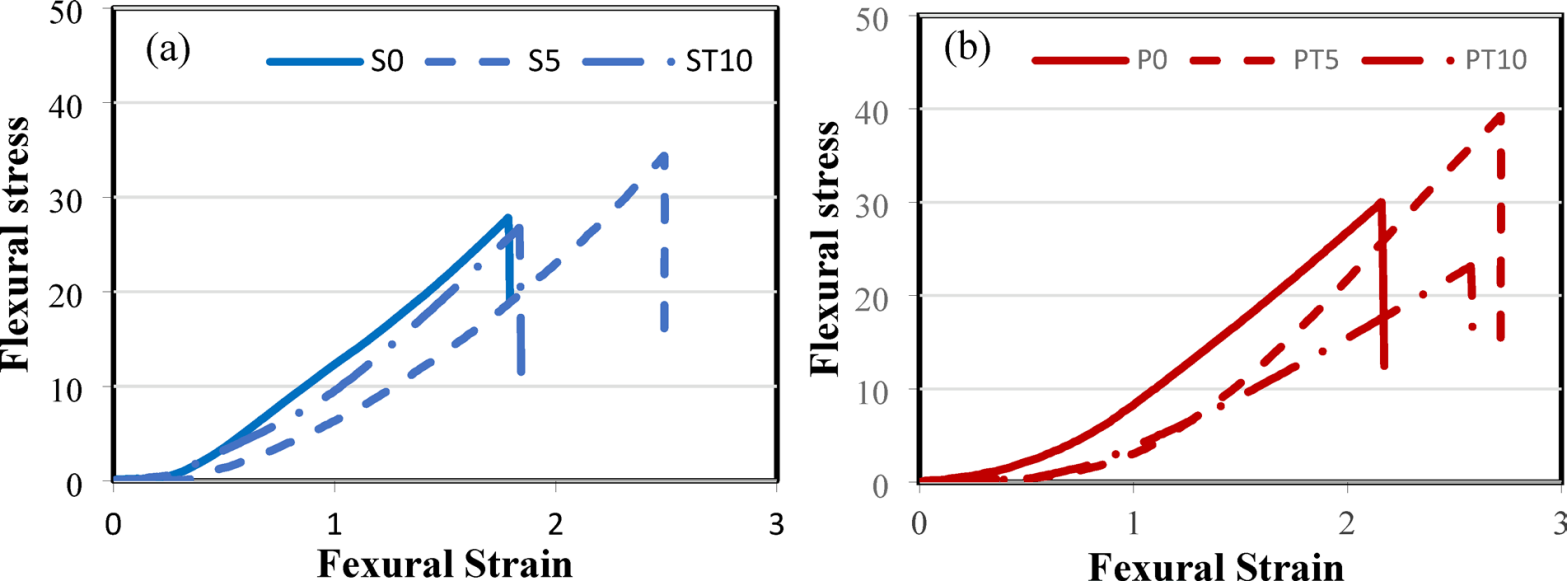
Flexural stress strain curve of (**a**) Swedish wood/epoxy composites, and (**b**) pitch pine wood /epoxy composites.

Figure 13 shows the flexural stress–strain curves of the wood–epoxy composites provide insight into their load-bearing capacity, and deformation behavior under bending test. The composites include untreated samples (S0 and P0) and alkali-treated samples at 5% (ST5 and PT5) and 10% (ST10 and PT10) concentrations**.** All specimens display an initial linear elastic area, representing elastic deformation under three-point bending. Al specimens exhibit a sudden stress drop at final failure, characteristic of flexural fracture. The 5% treated composites sustain the highest strain at peak stress, indicating improved flexural toughness and energy absorption capability. The 10% treated composites fail at lower stress and strain levels, demonstrating more brittle behavior. This supports the hypothesis that excessive alkali treatment compromises fiber structural integrity, leading to reduced toughness and strength despite potential improvements in surface adhesion.

**Fig. 14:**
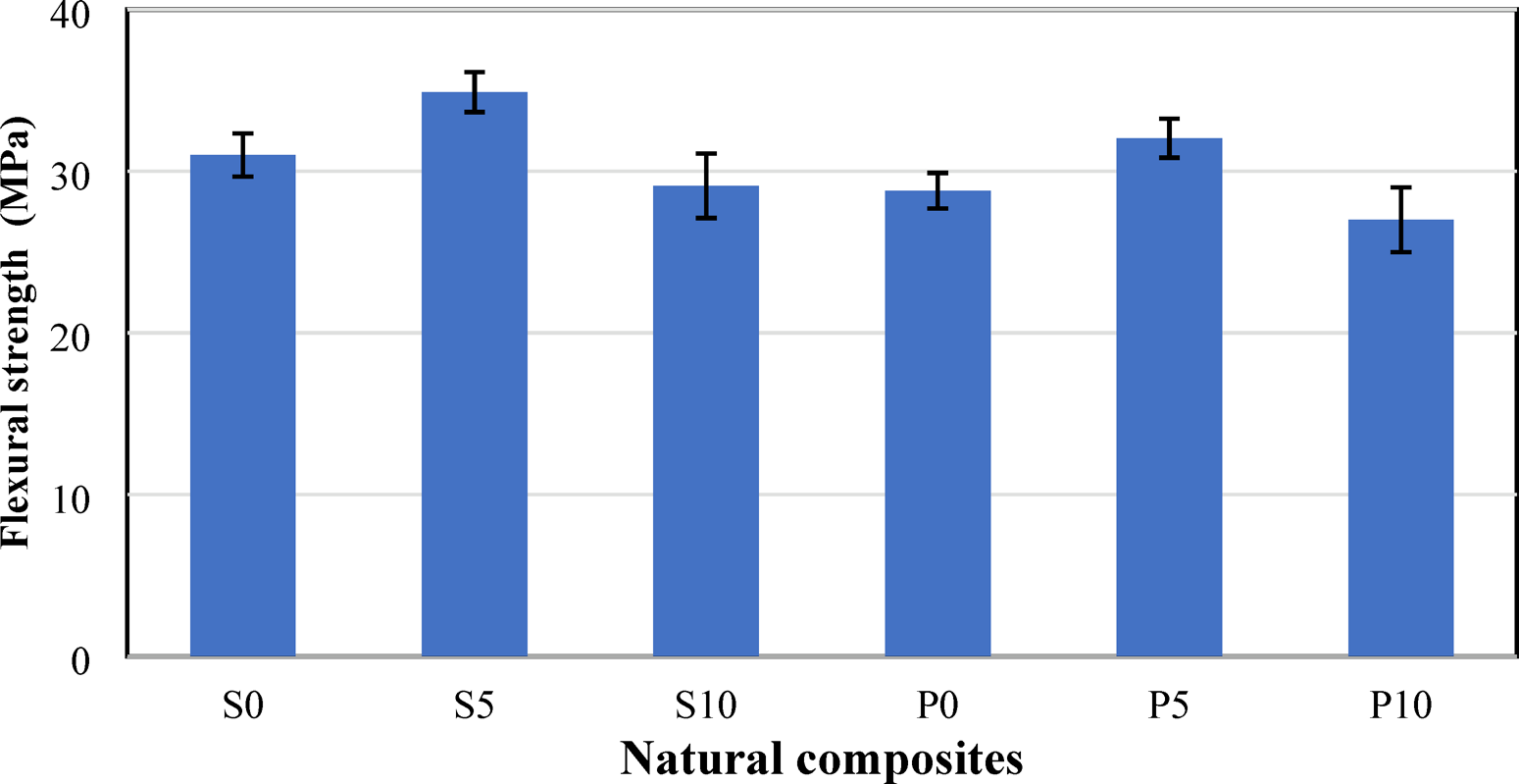
Flexural strength of treated and untreated wood/epoxy composites.

**Fig. 15:**
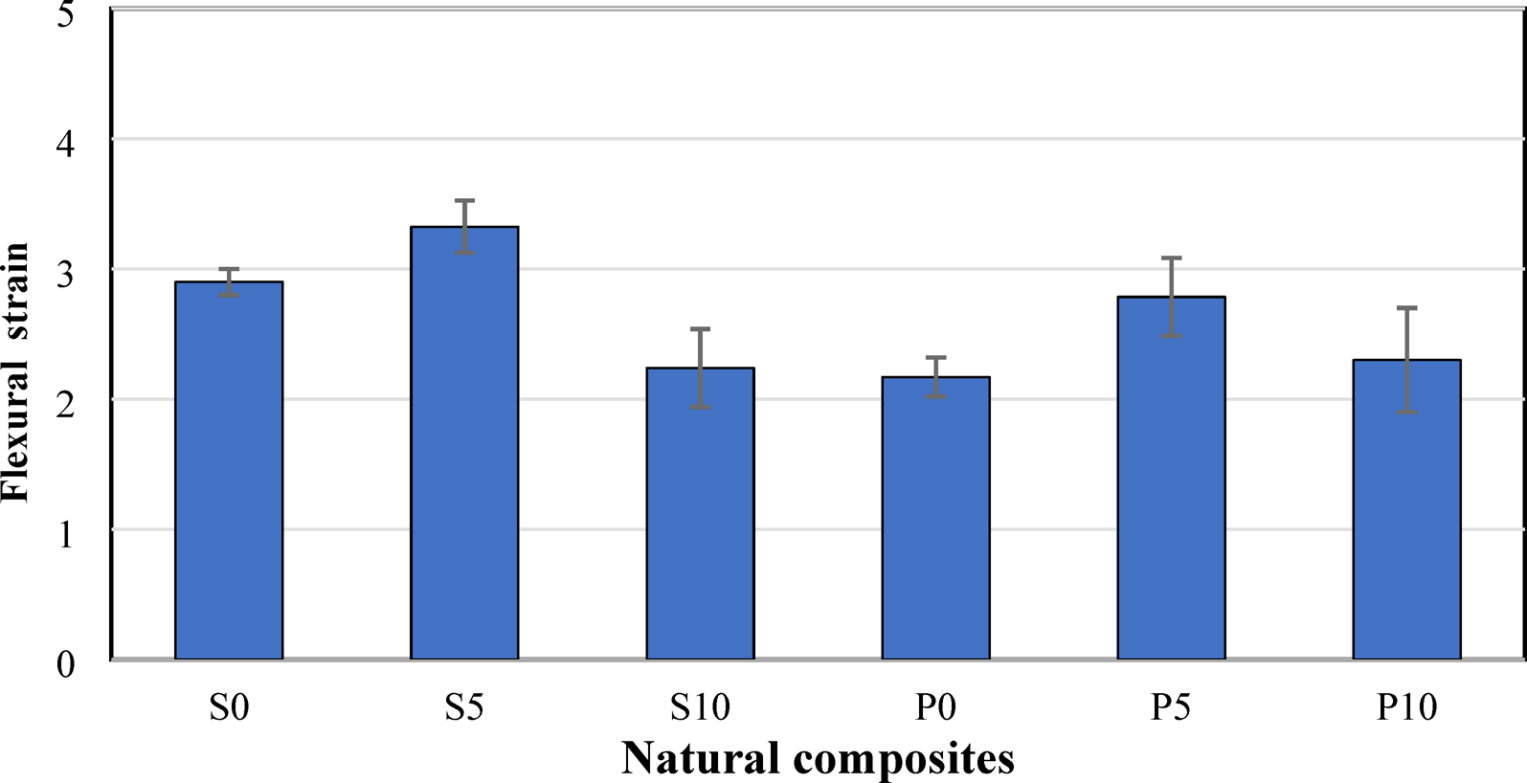
Flexural strain of treated and untreated wood/epoxy composites.

**Fig. 16:**
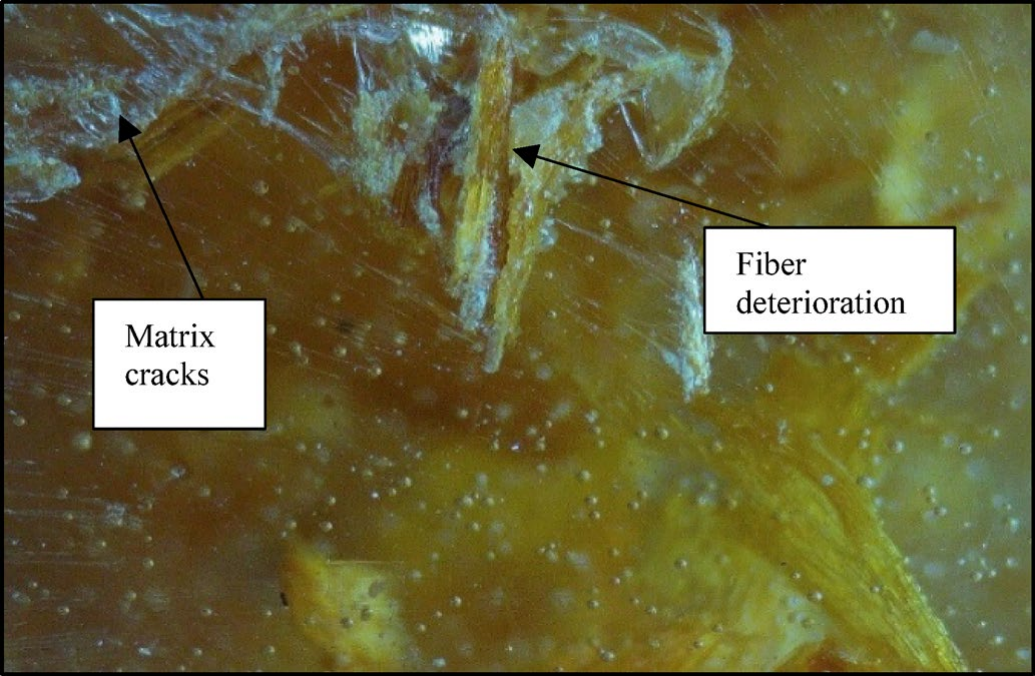
The flexural fracture surface for 10 wt% treated Swedish wood/epoxy specimens, ST10.

Figures 14 and 15 demonstrate the flexural behavior of natural composites. The efficient removal of hemicellulose, lignin, and surface contaminants without harming the structural integrity of the wood fibers is responsible for ST5’s exceptional performance. This leads to enhanced stress transfer between the fibers and the epoxy matrix, allowing the composite to bear higher flexural stresses. On the other hand, the decrease in flexural strength seen for ST10 indicates that fiber degradation, such as microfibril damage, severe delignification, or decreased fiber cross-section, is caused by an excessive alkali concentration. Premature failure under bending is the result of these processes, which weaken the fibers and decrease their capacity to support loads. Non-uniform fiber dispersion, which probably occurred from excessive alkali treatment was also indicated from Fig. 16. Over-treated fibers tend to agglomerate due to increased surface roughness and reduced fiber integrity, making consistent wetting and dispersion within the epoxy matrix challenging. Resin-rich zones display reduced stiffness and strength, while fiber clusters limit effective load transmission, favoring early fracture initiation under flexural loading. Furthermore, improper fiber distribution reduces the effectiveness of interfacial bonding, raising the risk of interlaminar failure, matrix cracking, and fiber pull-out during bending. Because of this, even with treated fibers present, the composite shows decreased flexural strength and stiffness.

The enhanced flexural performance of the 5% NaOH treated composites is strongly supported by the optical micrographs taken after the flexural tests. The micrographs reveal excellent interfacial bonding between the epoxy matrix and the treated wood fibers, along with a significant reduction in matrix cracks and micro-voids. This clean, intact interface confirms that the 5% alkaline treatment successfully cleaned the fiber surfaces and enhanced mechanical interlocking. Because the fiber-matrix bond is robust, stress is efficiently transferred across the interface during bending. This prevents the initiation of early matrix cracking and suppresses void-induced stress concentrations, allowing the composite to endure higher flexural stress and strain. Furthermore, the failure zone is characterized primarily by fiber breakage rather than fiber pull-out. This transition to fiber breakage confirms that the interfacial shear strength exceeds the tensile strength of the fibers themselves, proving that the 5% NaOH concentration achieves optimal interfacial adhesion without compromising fiber integrity^45,46^. Mechanical properties of vetiver fiber epoxy composites was studied as the hand layup technique was used to create the alkaline-treated VFs (vetiver fibers) and ER (epoxy resin) composites. The resulting composites demonstrated uniform adhesion and distribution between VFs and ER. After the VFs were alkali-treated in a 0.5 mol/L NaOH solution for four hours, the VF/ER composites’ maximum mechanical properties-impact, tensile, compressive, and flexural strengths were achieved. The mechanical strengths of the VF/ER composites dropped and the VFs were damaged at higher NaOH concentrations. The VFs, however, have received treatment. The mechanical properties of composite materials are still improved more than those of untreated VFs. However, it needs to be at the proper level so that it can be used with other materials for reinforcement^47^.

**Fig. 17:**
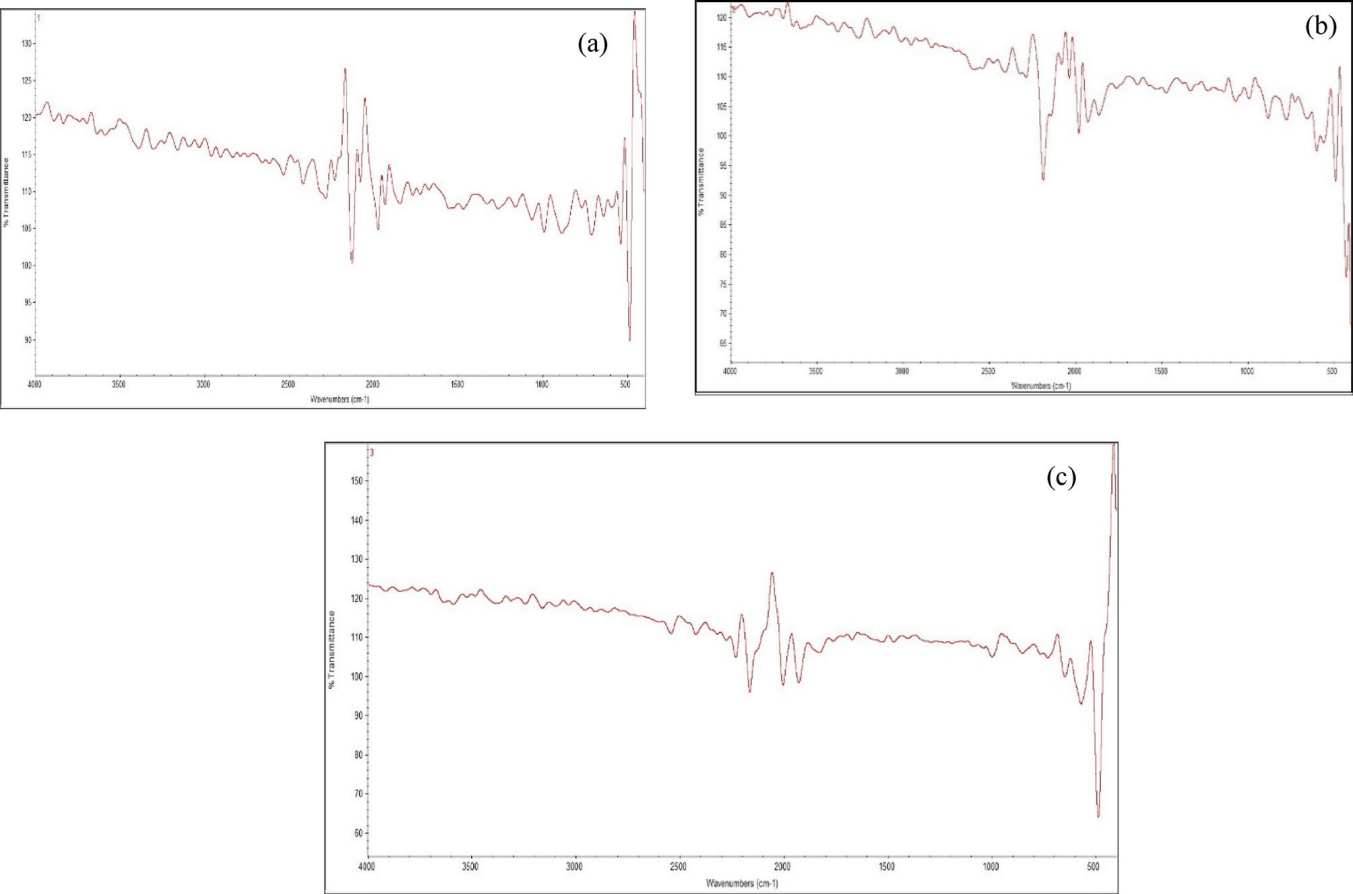
FTIR for wood/epoxy specimens (**a**) S0, (**b**) S5, and (**c**) S10.

The FTIR spectra of the Swedish wood/epoxy composites that were untreated (S0) and alkali-treated (S5 and S10) are displayed in Fig. 17a, b, and c, respectively. Slight changes in the strength of numerous absorption bands were found after alkali treatment, indicating the removal of surface contaminants, hemicellulose, and part of the lignin from the wood fibers. In line with its enhanced tensile, flexural, and impact qualities, the S5 specimen showed the best spectrum features, indicating successful surface alteration while maintaining the cellulose structure. The S10 specimen, on the other hand, displayed more noticeable spectrum alterations, suggesting that extensive removal of fiber constituents may have led to fiber breakdown and the ensuing decrease in mechanical performance.

#### Impact test

**Fig. 18:**
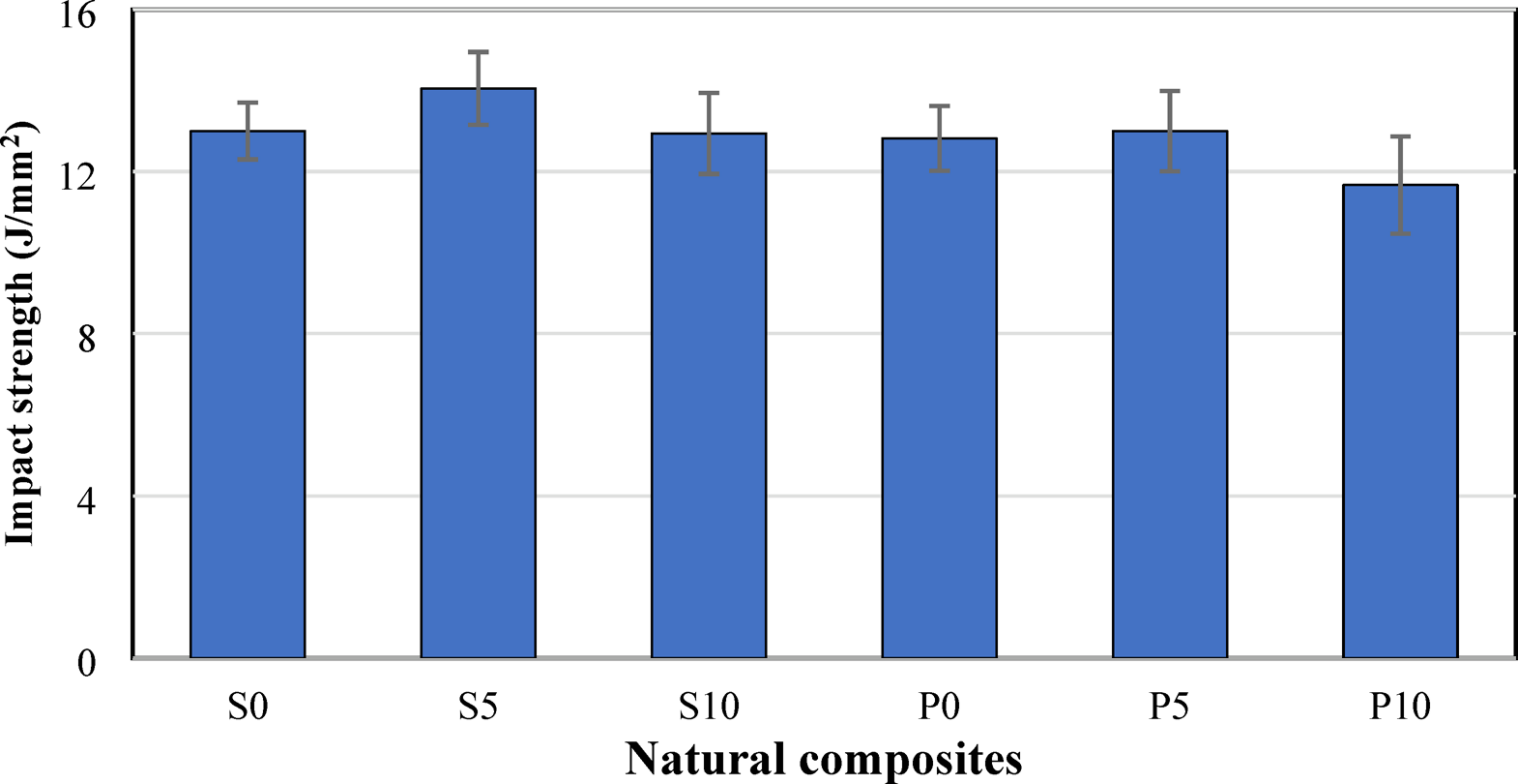
Impact strength of treated and untreated wood/epoxy composites.

The energy absorption properties of untreated and chemically treated wood composites were assessed through an impact analysis. The untreated composites S0 and P0 show moderate impact strength levels of roughly 0.31 J/mm^2^ and 0.32 J/mm^2^, respectively as shown in Fig. 18. These results suggest inadequate energy absorption capability due to weak fiber–matrix interfacial adhesion, which favors brittle fracture and rapid crack propagation under impact loading. The 5% alkali-treated composites, ST5 and PT5, display the highest impact strength among all specimens, with ST5 showing the largest value (0.367 J/mm^2^). The improvement in impact resistance can be attributed to better interfacial adhesion resulting from moderate alkali treatment. Improved bonding permits effective stress transfer and enhances energy-dissipating mechanisms such as fiber debonding, pull-out, and controlled matrix cracking, hence boosting the toughness of the composites^48^.

The impact strength of the 10% alkali-treated composites, ST10 and PT10, is lower than that of the 5% treated samples, with PT10 having the lowest value (0.29 J/mm^2^). Excessive alkali treatment, which can weaken fiber structure and result in uneven fiber dispersion as seen in optical microscopy, is linked to this decline. The composite’s capacity to absorb and disperse impact energy is lowered by fiber degradation and aggregation, which causes early fracture under dynamic loading.

#### Hardness test

**Fig. 19:**
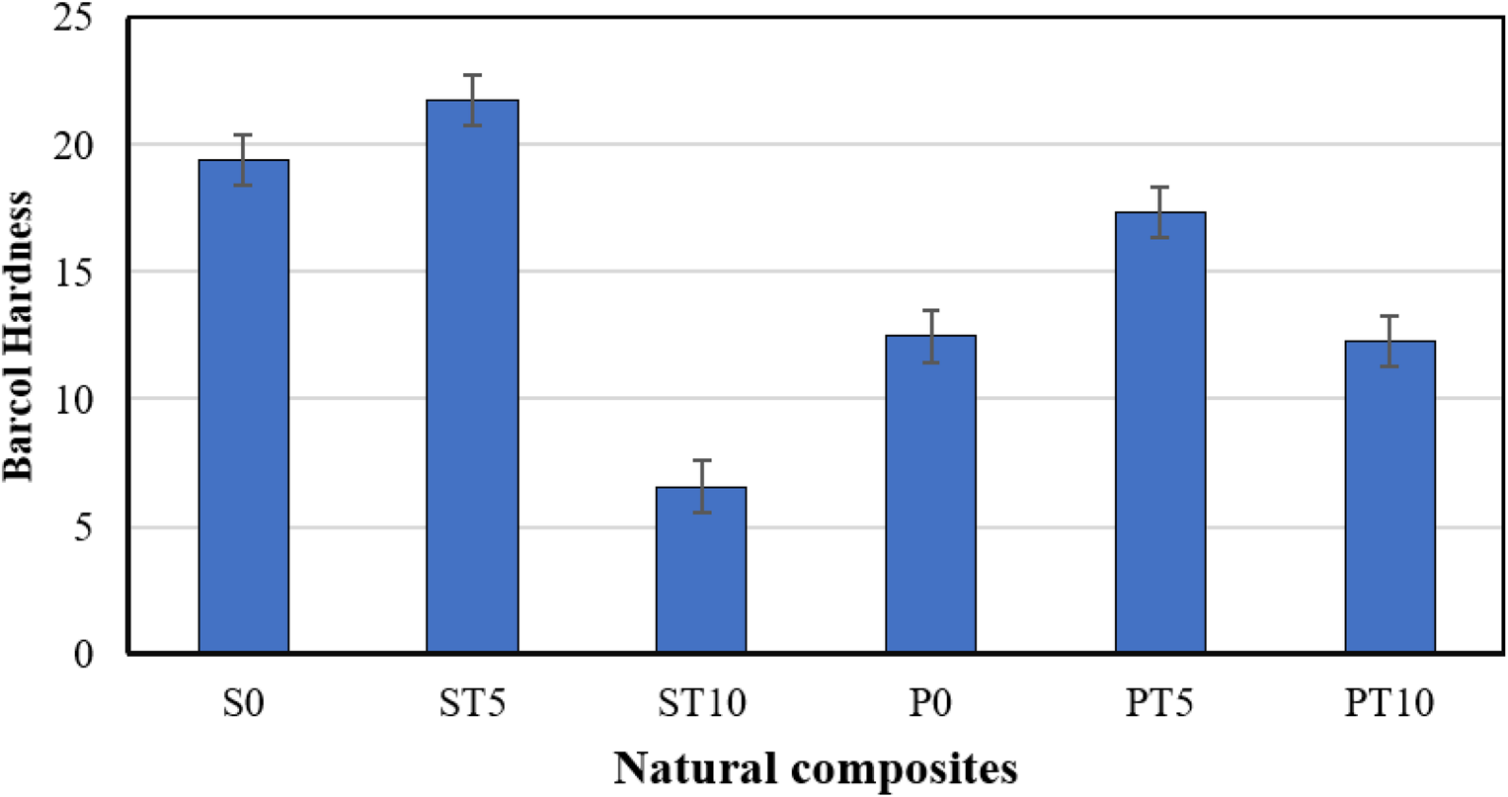
Hardness of treated and untreated wood/epoxy composites.

The surface hardness of the wood-epoxy composites was found to be significantly influenced by the concentration of the alkali treatment as shown in Fig. 19. An initial increase in hardness was observed for the 5% NaOH treated composites compared to the untreated specimens, which can be attributed to the removal of surface impurities and the resulting increase in interfacial packing density between the fiber and the epoxy matrix. However, a subsequent decline in hardness was recorded as the treatment level reached 10%. This reversal is likely due to excessive delignification and the degradation of the fiber’s structural integrity, which reduces the composite’s resistance to localized surface deformation. Figure 19 illustrates that So is better than PW in hardness properties.

#### Density of the natural composites

**Fig. 20:**
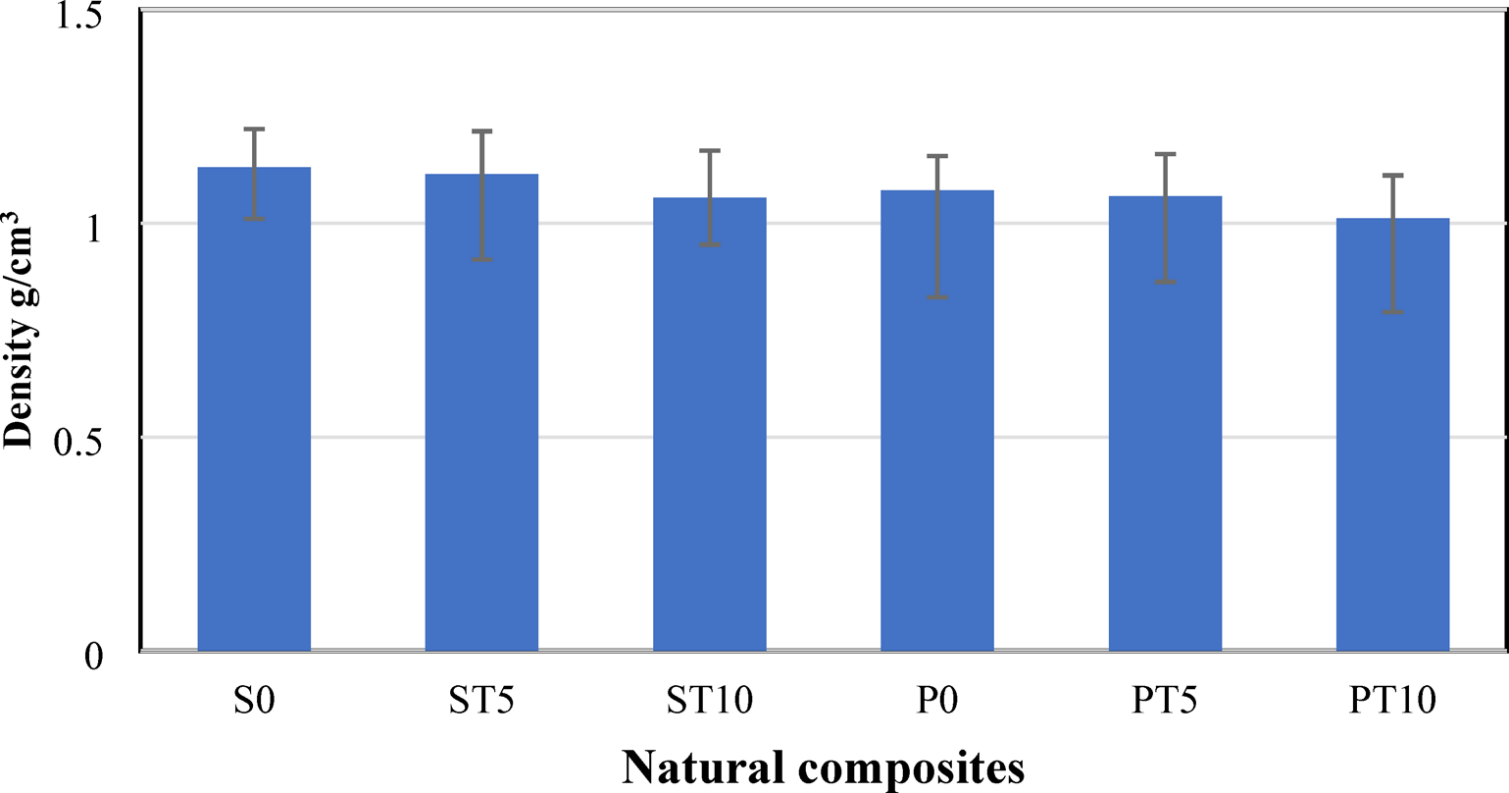
Density of treated and untreated wood/epoxy composites.

The mechanical and structural efficiency of wood-epoxy composites is significantly influenced by density, a basic physical parameter. The bulk density of the manufactured specimens is evaluated in this part in order to comprehend the microstructural modifications brought about by chemical modification and nanomaterial reinforcement. As shown in Fig. 20 for both Swedish wood and Pitch Pine wood composites, chemical treatment leads to drop in density compared to their untreated counterparts (S0 and P0) as the removal of surface impurities, hemicellulose, waxes, and part of the lignin during the alkali treatment, resulting in a lower overall mass of the reinforcing fibers^49,50^. After alkali treatment, the density of both Swedish wood and Pitch Pine wood composites slightly decreased, but this did not negatively impact their mechanical performance. The treated fiber surfaces showed better cleanliness and interfacial adhesion with the epoxy matrix at the ideal treatment concentration of 5 wt% NaOH, which encouraged more effective stress transmission and thus enhanced the tensile, flexural, hardness and impact properties. Therefore, rather than the breakdown of the reinforcing cellulose, the modest drop in density is linked to the removal of non-load-bearing elements. On the other hand, even while the density continued to decrease, treatment with 10 wt% NaOH resulted in significant fiber breakdown and a decline in mechanical performance. This behavior confirms that while alkali treatment inherently lowers fiber density, a 5% NaOH concentration represents the ideal thermodynamic window to maximize surface hardness through localized interfacial compacting without inducing core structural failure^47^.

#### Thermogravimetric analysis (TGA)

**Fig. 21:**
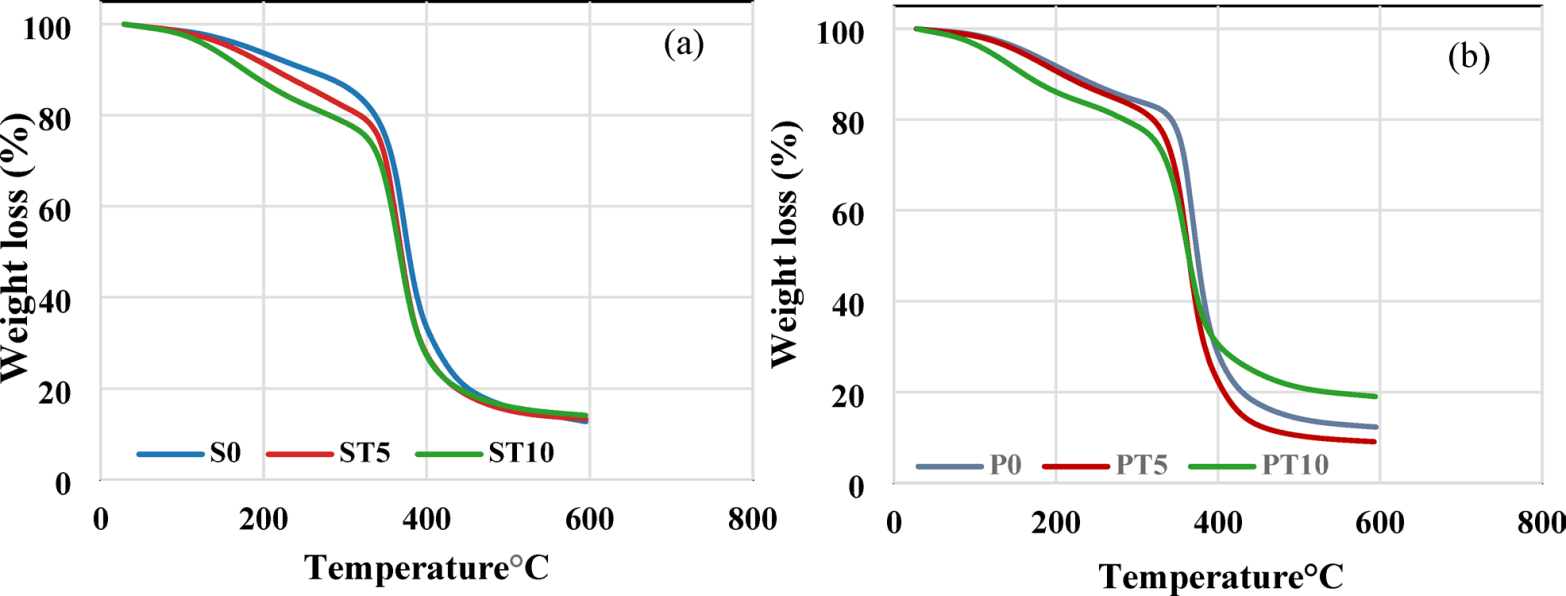
Thermogravimetric Analysis (TGA) for untreated and treated wood epoxy composites (**a**) Swedish wood (**b**) Pitch Pine wood.

Figure 21a, b shows the thermal degradation behavior of the composites was analyzed using thermogravimetric analysis (TGA) of Swedish and Pitch Pine wood, respectively. The results reveal a clear multi-stage weight loss profile, where the untreated composites, S0 and P0, exhibit the highest initial thermal stability. Upon introduction of chemical modifications, the decomposition curves of the 5% and 10% alkali-treated samples for both Swedish and Pitch Pine wood systematically slightly shift toward lower temperatures, signaling a reduction in overall thermal resistance. Alkali treatment with 5% successfully extracts amorphous components like hemicellulose and surface waxes, establishing an optimal, rough topography that yields good mechanical interlocking with the epoxy resin matrix. In comparison to the untreated specimens, the 5 wt% NaOH-treated composites, both Swedish and Pitch Pine wood, showed a slight shift of the main degradation region toward higher temperatures, indicating improved thermal stability due to improved fiber–matrix interfacial bonding and the removal of thermally unstable constituents. The 10 wt% NaOH-treated composites for both Swedish and Pitch Pine wood, on the other hand, displayed earlier thermal degradation, indicating that the excessive alkali treatment reduced the thermal stability by partially damaging the fiber structure. The mechanical results, which showed that the 5 wt% NaOH treatment offered the best balance between surface modification and fiber integrity, are in line with these findings.

**Fig. 22:**
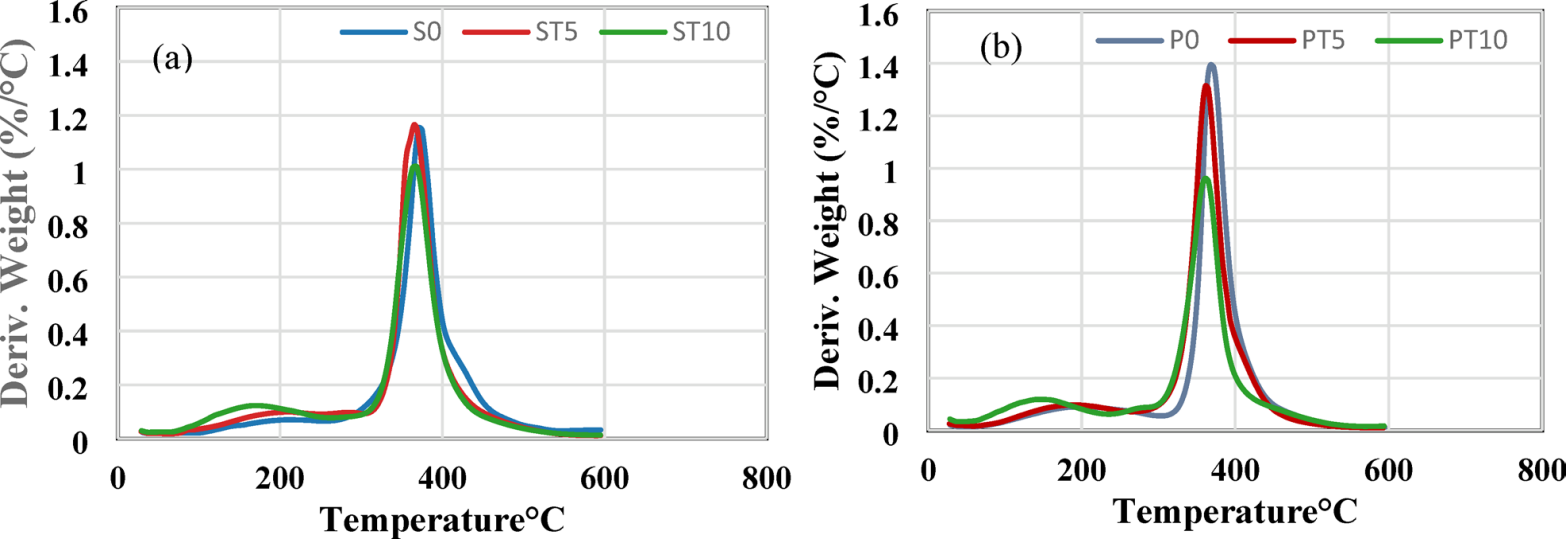
Derivative Thermogravimetric analysis for untreated and treated wood epoxy composites (**a**) Swedish wood (**b**) Pitch Pine wood.

The Derivative Thermogravimetric analysis (DTG) curves of the alkali-treated and untreated Swedish and pitch pine wood/epoxy composites are shown in Fig. 22 (a, b), respectively. With a minor shift of the maximum degradation peak toward higher temperatures, the 5 wt% NaOH-treated both swedish and pitch pine wood/epoxy composites showed degradation behavior very similar to that of the untreated composites. This suggests that the treatment successfully altered the fiber surface without sacrificing its thermal stability. The 10 wt% NaOH-treated composites, ST10 and PT10, on the other hand, displayed a shift of the degradation peak toward lower temperatures, indicating that the wood fibers were partially damaged and their thermal stability was diminished by the excessive alkali treatment. These findings support the mechanical test results, demonstrating that 5 wt% NaOH offers the best possible compromise between surface modification, fiber integrity, and thermal performance.

### Mechanical properties of graphene nanoplatelet/wood epoxy composites

#### Tensile test

**Fig. 23:**
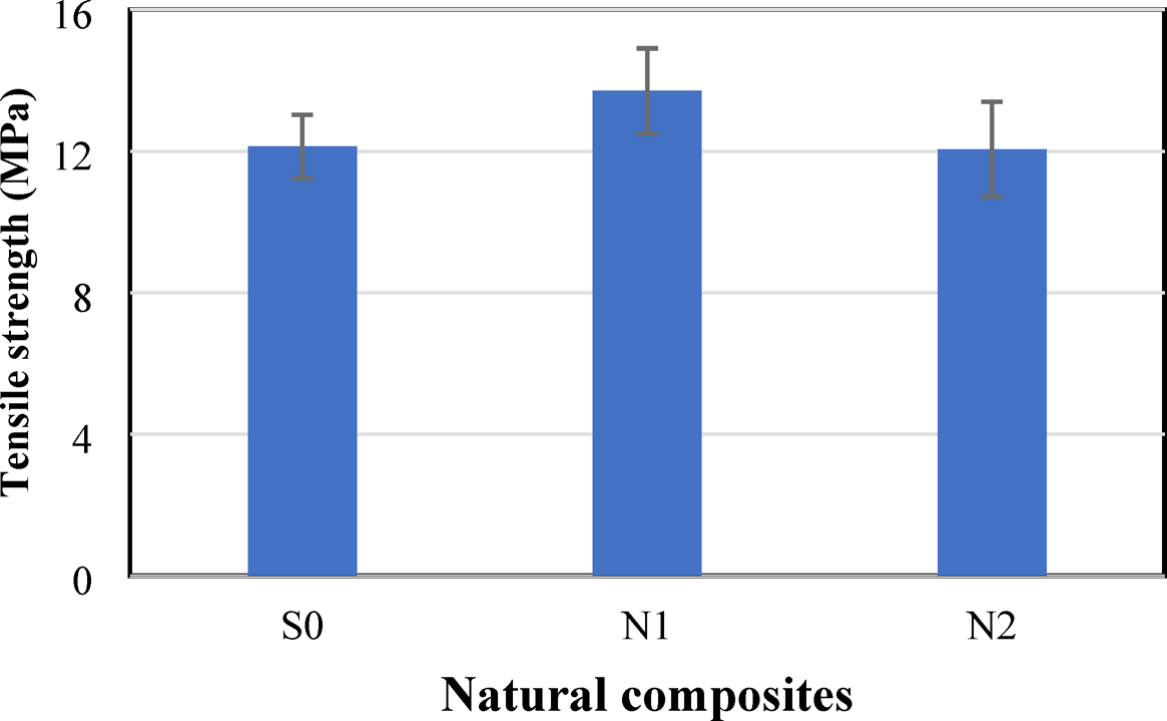
The tensile strength of unfilled wood epoxy composites and nano filled wood composites.

Figure 23 shows the tensile strength of unfilled wood epoxy composites and nano filled wood composites. The tensile strength of the unfilled wood-fiber epoxy composites initially increased upon the addition of 0.125 wt% graphene nanofillers. This enhancement is likely due to the high aspect ratio of graphene and its ability to facilitate efficient stress transfer within the epoxy matrix. However, when the graphene content was increased to 0.25 wt%, a subsequent decrease in tensile strength was observed. This reduction is typically attributed to filler agglomeration and the formation of stress concentration sites at higher loading levels, which can lead to premature structural failure. Fillers usually enable composites to achieve better mechanical and thermal properties that fiber-reinforced composites without fillers would not be able to. Natural or synthetic fillers are typically reinforced in the nanoscale according to the requirements in order to form nanocomposites^51^.

A study was carried out to determine the effect of graphene nanoparticle (GNPs) reinforcement on the mechanical properties of glass fiber reinforced polymer (GFRP), carbon fiber reinforced polymer (CFRP) and aramid fiber reinforced polymer (AFRP) composites commonly used in the space and defense industry. The hot-pressing method was used to create GFRP, CFRP, and AFRP composites. Also, by adding 0.1%, 0.2%, and 0.3% GNPs into these fiber-reinforced composites, hybrid fiber composites were created. Tensile strength and modulus of elasticity were found to increase when 0.2% GNPs was added to GFRP and CFRP composites. Nevertheless, the tensile strength of AFRP composites was unaffected by the addition of 0.2 wt% GNPs; instead, it somewhat decreased the tensile strength while raising the modulus of elasticity^52^

**Fig. 24:**
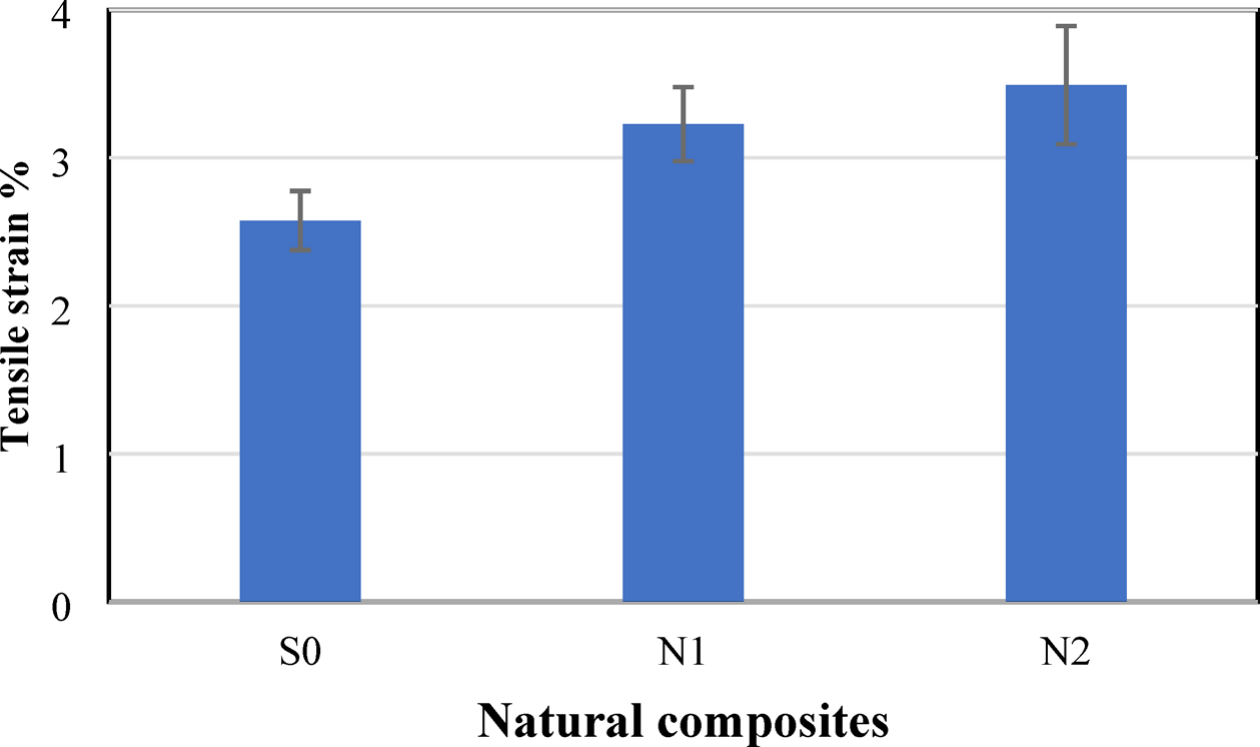
The tensile strain of unfilled wood epoxy composites and nano filled wood composites.

In contrast, a clear increase in tensile strain (elongation at break) was evident at both concentrations as shown in Fig. 24 This suggests that the presence of graphene may induce a ductilizing effect, possibly through mechanisms such as inter-particle gliding or the promotion of plastic deformation within the epoxy matrix. These findings indicate that while 0.125 wt% may be the optimal concentration for maximizing strength, the inclusion of graphene consistently improves the material’s overall deformability and ductility. Similar results were obtained by Lapčík, et al.^53^. An increasing ductility of the studied nanocomposites was found in the concentration range of 0–1 wt% GNP filler concentration, as reflected in the samples’ increased elongation at break. This type of behavior was interpreted by the inter-particle gliding effect of the individual GNP nanoparticles dispersed in the complex epoxy resin matrix.

**Fig. 25:**
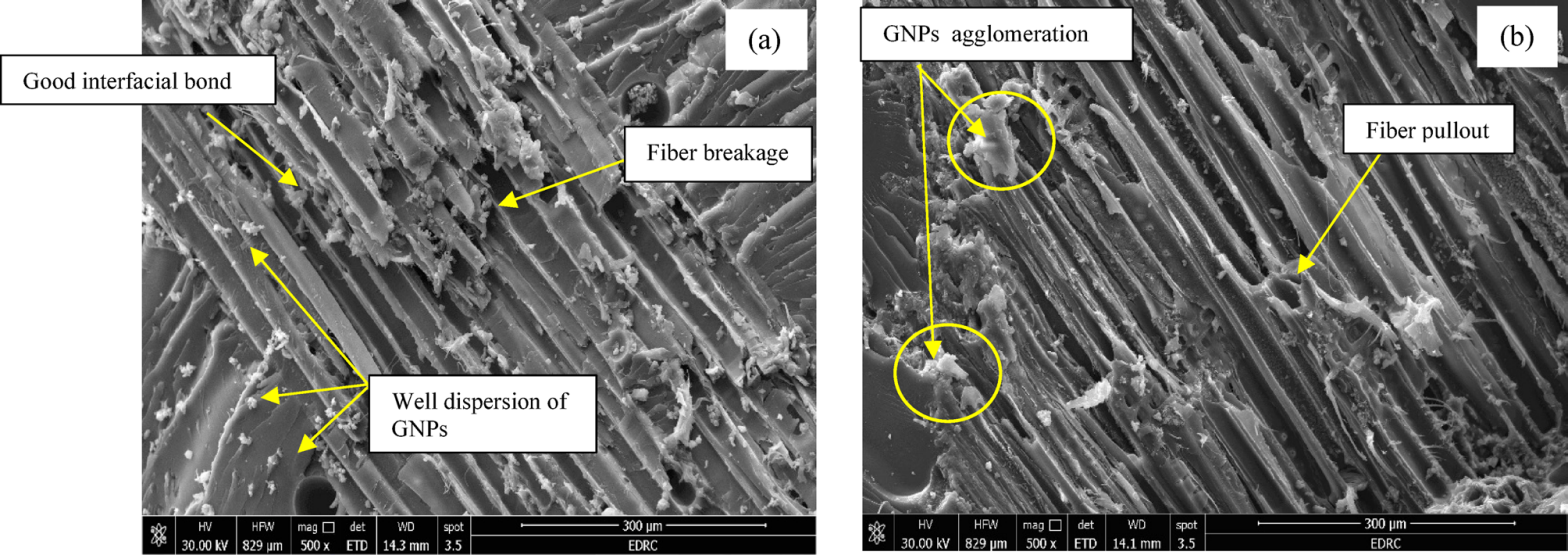
SEM images for fracture surfaces for (**a**) N1 and (**b**) N2 specimens after subjecting to tensile loading.

Figure 25 discusses the fracture surfaces of GNPs incorporated wood epoxy composites subjected to tensile loading. The fracture morphology in Fig. 25a reveals a relatively well-integrated microstructure with well-bonded wood fibers tightly wrapped by the epoxy matrix. This directly supports the claim that the high aspect ratio of graphene at this optimal concentration ensures a uniform stress transfer path across the interface. The failure shows a clean break across both the matrix and the embedded wood fibers simultaneously, which is characteristic of the enhanced tensile strength mentioned. On the other hand the fracture surface in Fig. 25b appears highly irregular, characterized by visible graphene cluster formations and localized rough patches. These clusters correspond directly to filler agglomeration. They act as internal defects and critical stress concentration points that initiate microcracks prematurely, ultimately lowering the overall tensile strength. The weak interfacial bonding caused by these agglomerations leads to fiber pull-out as a prominent failure mode. Instead of breaking simultaneously with the matrix, the wood fibers separate and slide out of the epoxy completely. This leaves behind hollow cylindrical voids and detached fiber strands across the topography^54^.

#### Flexural test

**Fig. 26:**
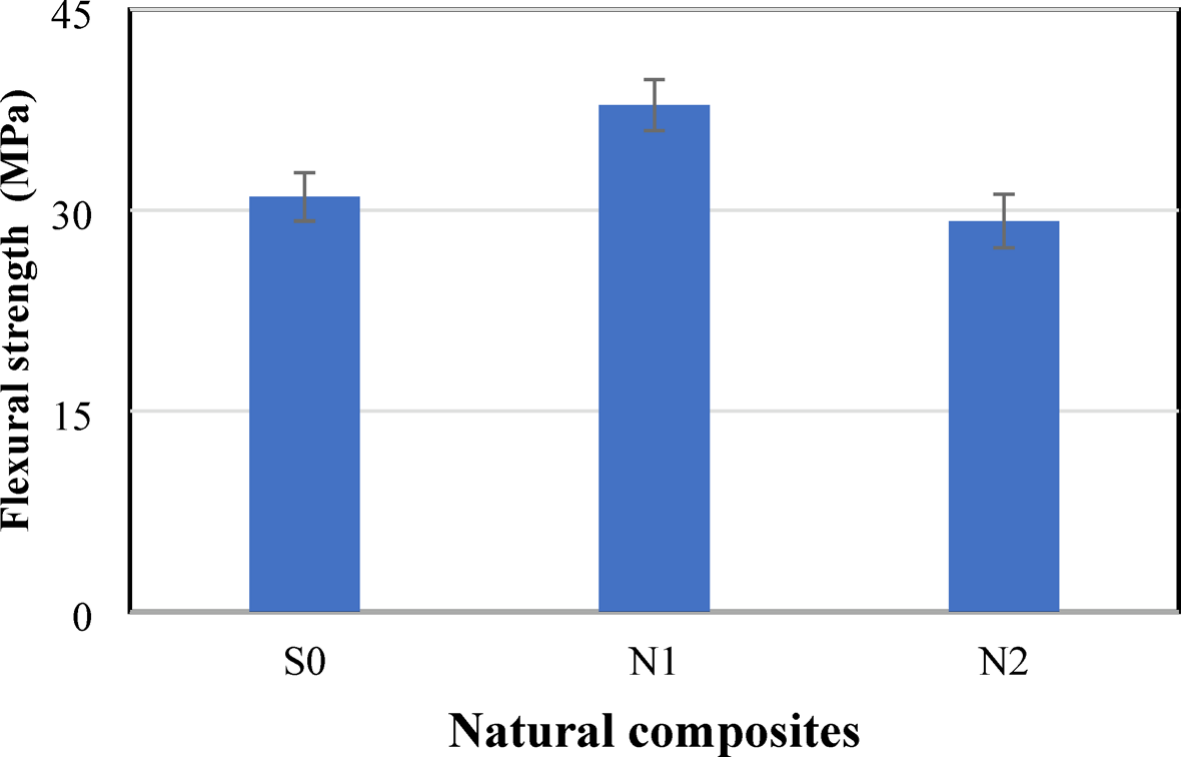
The flexural strength of unfilled wood epoxy composites and nano filled wood composites.

As shown in Fig. 26, the flexural strength of the wood-epoxy composite initially increased with the addition of 0.125 wt% graphene nanoplatelets, followed by a notable decrease as the GNPs concentration was raised to 0.25 wt%. This biphasic mechanical response is attributed to the transition from a dispersed reinforcement state to a state dominated by filler agglomeration.

Enhancement at 0.125 wt% may be attributed to that the GNPs are generally well-dispersed, allowing for efficient stress transfer from the polymer matrix to the high strength nanoplatelets. The large surface area of individual GNPs promotes toughening mechanisms such as crack pinning and crack deflection, which effectively hinder crack propagation under bending loads. Degradation at 0.25 wt% may be attributed to that the reduction in strength at higher concentrations occurs when the filler content exceeds the dispersion limit of the matrix. Above this threshold, van der Waals forces cause the nanoplatelets to form agglomerates, which act as localized stress concentrators rather than reinforcements. Furthermore, these clusters can entrap air and inhibit proper resin wetting, creating voids and weakening the interfacial bonding between the wood fibers and the epoxy matrix.

A similar trend of variation in flexural strength was also observed in the case of epoxy/graphene nanocomposite processed by combination method of mechanical mixing and bath sonication^55^. It was found that the flexural strength of epoxy/graphene nanocomposites processed by isolation method of high-speed mechanical mixing first increased with increasing graphene loading up to 0.2 wt% followed by a relative decrease with further increase of graphene loading to 1.0 wt%.

**Fig. 27:**
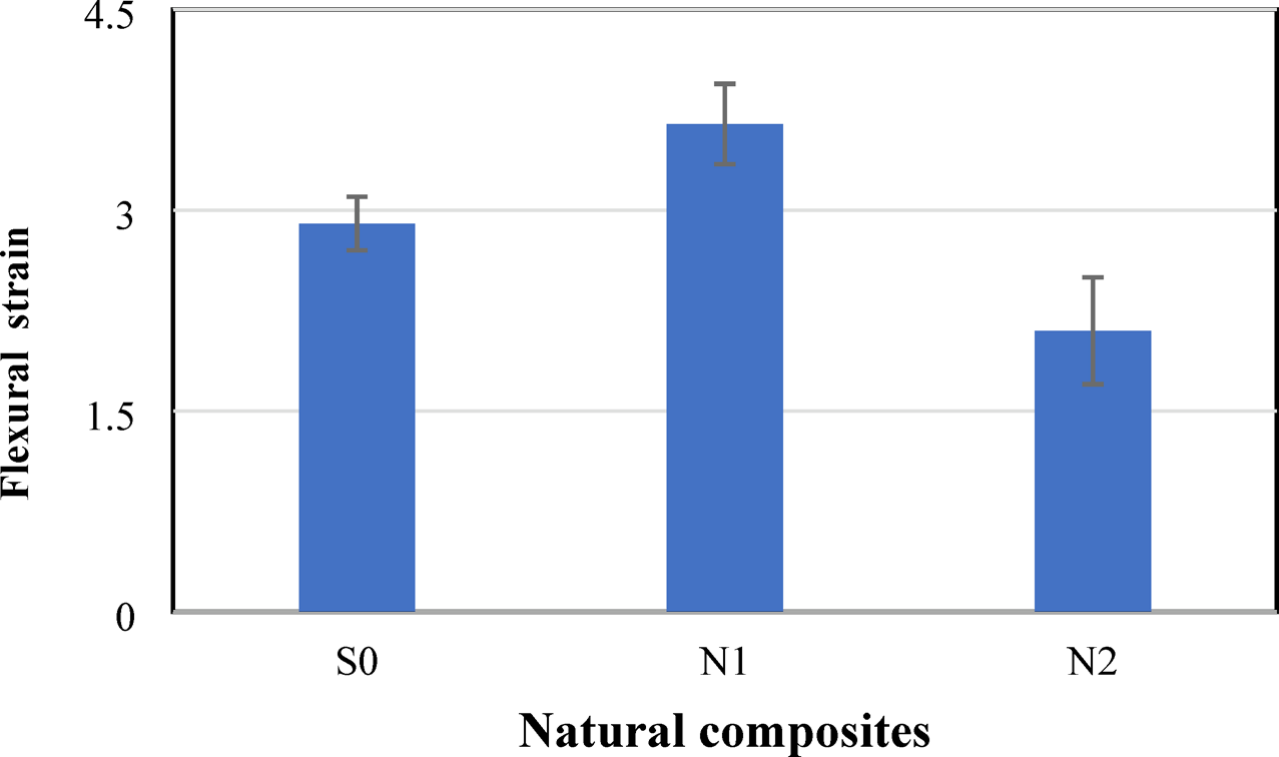
The flexural strain of unfilled wood epoxy composites and nanofilled wood composites.

Composites with 0.125% GNPs have the highest flexural strain, at very low concentrations. Well dispersed GNPs can enhance the composite’s flexibility and strain-to-failure by promoting micro-plastic deformation around the nanoplatelets as shown in Fig. 27. The high aspect ratio and energy-dissipating mechanisms of graphene, such as sheet sliding and bending, allow the matrix to undergo greater deformation before total fracture occurs.

**Fig. 28:**
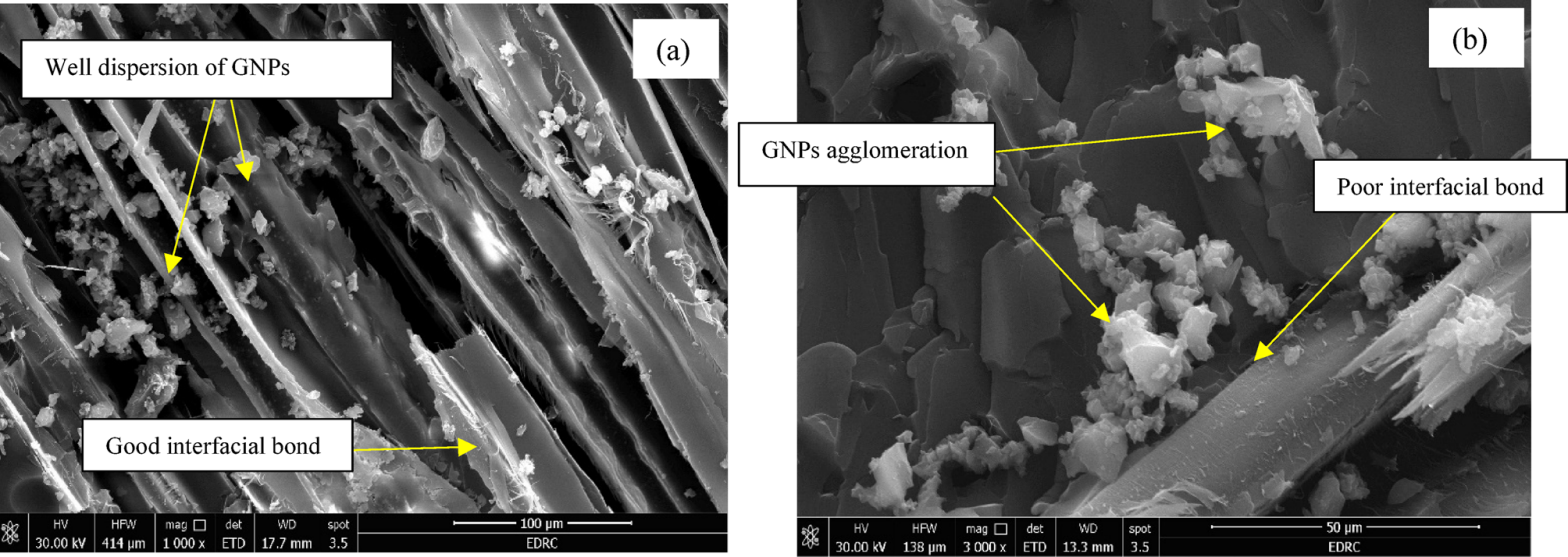
SEM images for fracture surfaces for (**a**) N1 and (**b**) N2 specimens after subjecting to flexural loading.

Figure 28 shows the fracture surface for GNPs incorporated wood epoxy composites subjected to flexural loading. Figure 28a shows the fracture surface of the optimal N1 specimen that highlights a clean, uniformly wet topography where the epoxy matrix remains firmly adhered along the longitudinal ridges of the wood fibers, promoting crack pinning and deflection that enhance bending resistance. In contrast, Fig. 28b for N2 reveals massive, multi-layered graphene nanoplatelet clusters resting directly on and between the fiber pathways. These severe agglomerations physically block liquid resin wetting, leaving behind bare fiber segments, micro-voids, and delaminated interfaces that act as critical bending stress concentrators. Ultimately, both loading conditions confirm that while a low filler content optimizes interfacial cohesion and stress transfer, exceeding the dispersion limit introduces cluster defects that accelerate failure via fiber pull-out under tension and via non-wetted interface delamination under flexure.

#### Impact test

**Fig. 29:**
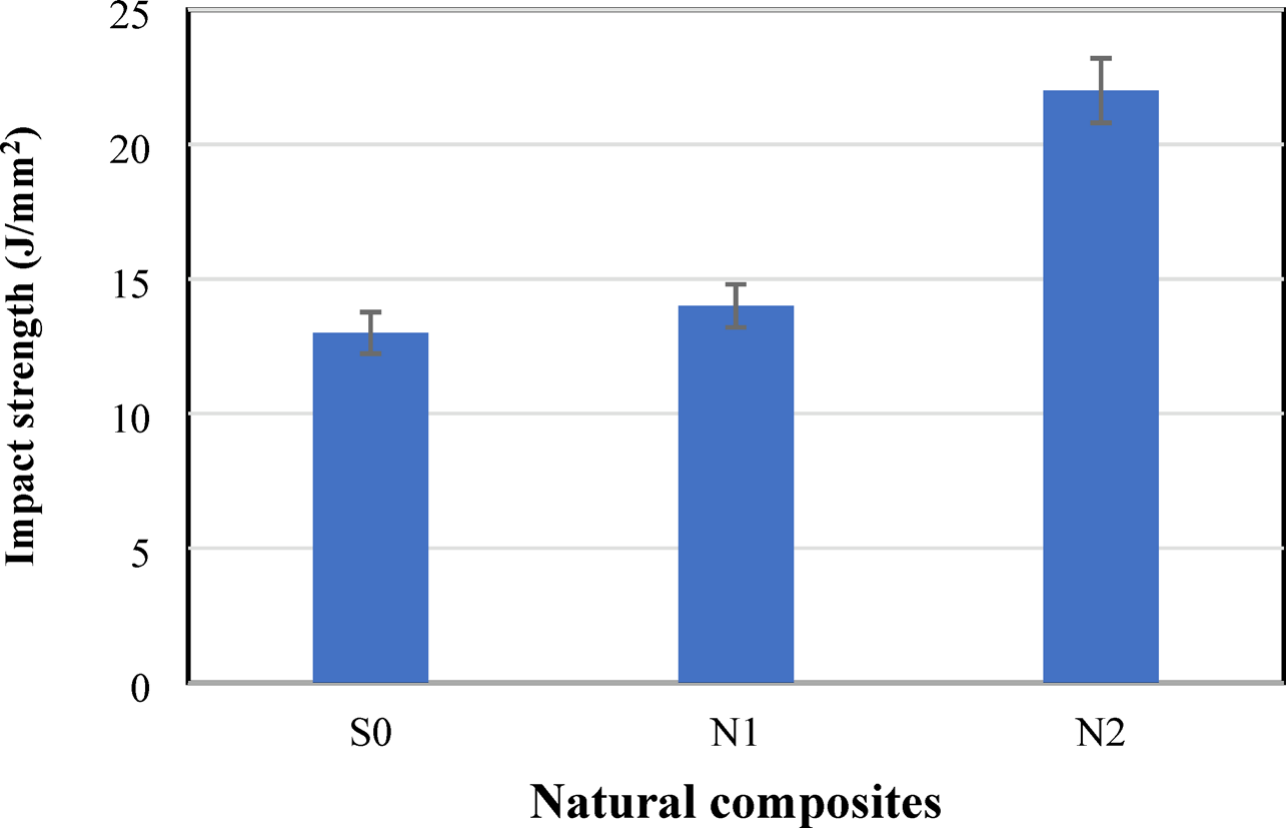
The impact strength of unfilled wood epoxy composites and nano filled wood composites.

As shown in Fig. 29, the impact strength of the wood-epoxy composite exhibited a marginal increase upon the addition of 0.125 wt% graphene nanoplatelets. However, a substantial enhancement in impact resistance was recorded at a loading of 0.25 wt%, resulting in a notable 64% increase in impact energy compared to the baseline composite. The increase in impact energy was attributed to the perfect adhesion between the fabrics and matrices via GNPs, providing more energy absorption capability and improved properties compared to other samples^56^. The effect of GNPs levels on absorbed energy of composites was studied and it was found that impact energy increased a bit with the addition of GNPs by 0.1%, then this increase was continued with more addition of GNPs to 0.25% which resulted in highest improving of absorbed energy^57^.

#### Density test

**Fig. 30:**
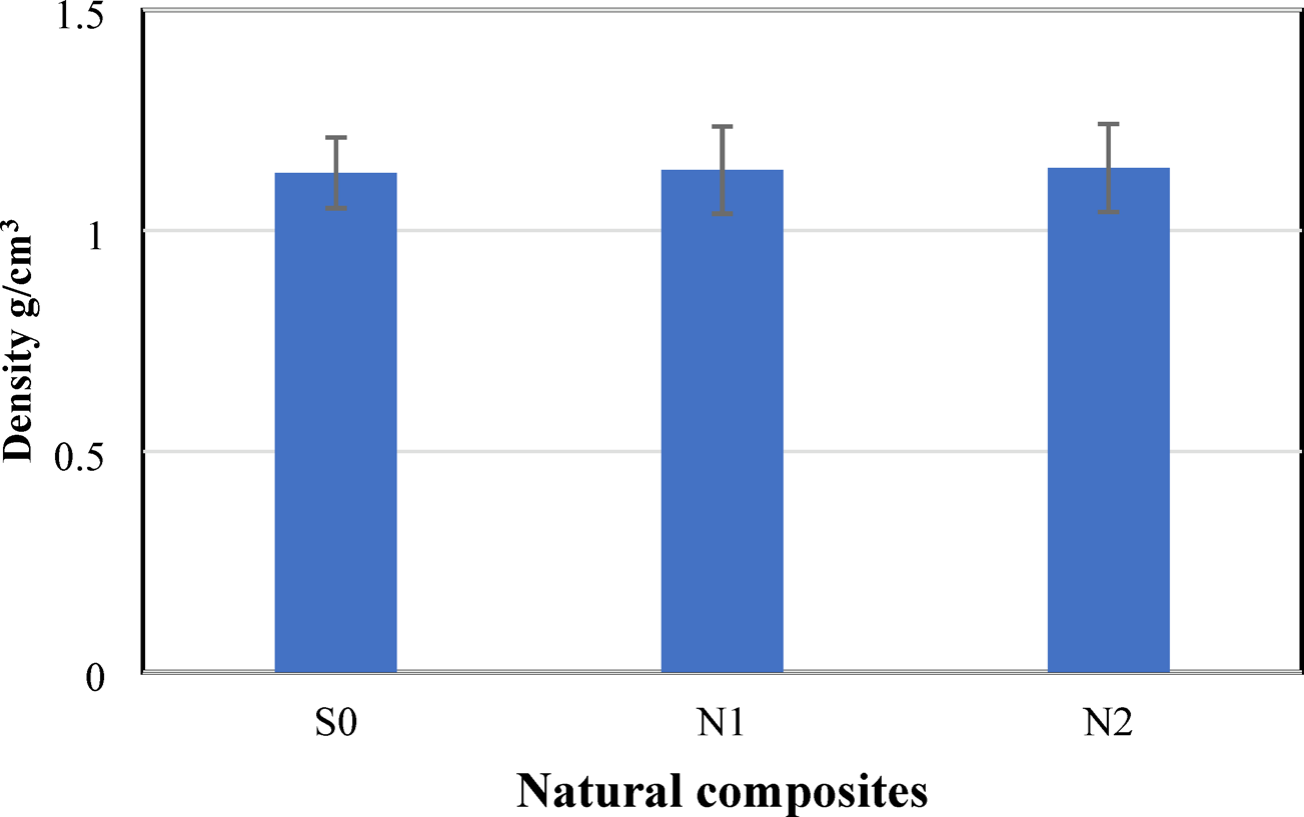
Density of unfilled wood epoxy composites and nano filled wood composites.

The addition of GNPs to the untreated wood composites results in the highest overall densities displayed on the chart. Introducing 0.125% GNPs (N1) raises the density above the untreated Swedish wood baseline. Doubling the concentration to 0.25% GNPs (N2) causes a further, slight incremental increase in density. When compared to porous wood fibers, graphene nanoplatelets have a substantially greater inherent material density. Furthermore, nano-fillers have a potential to occupy micro-voids in the epoxy matrix, increasing the overall density and packing the composite structure more tightly^58^ (Fig. 30)

#### Hardness test

**Fig. 31:**
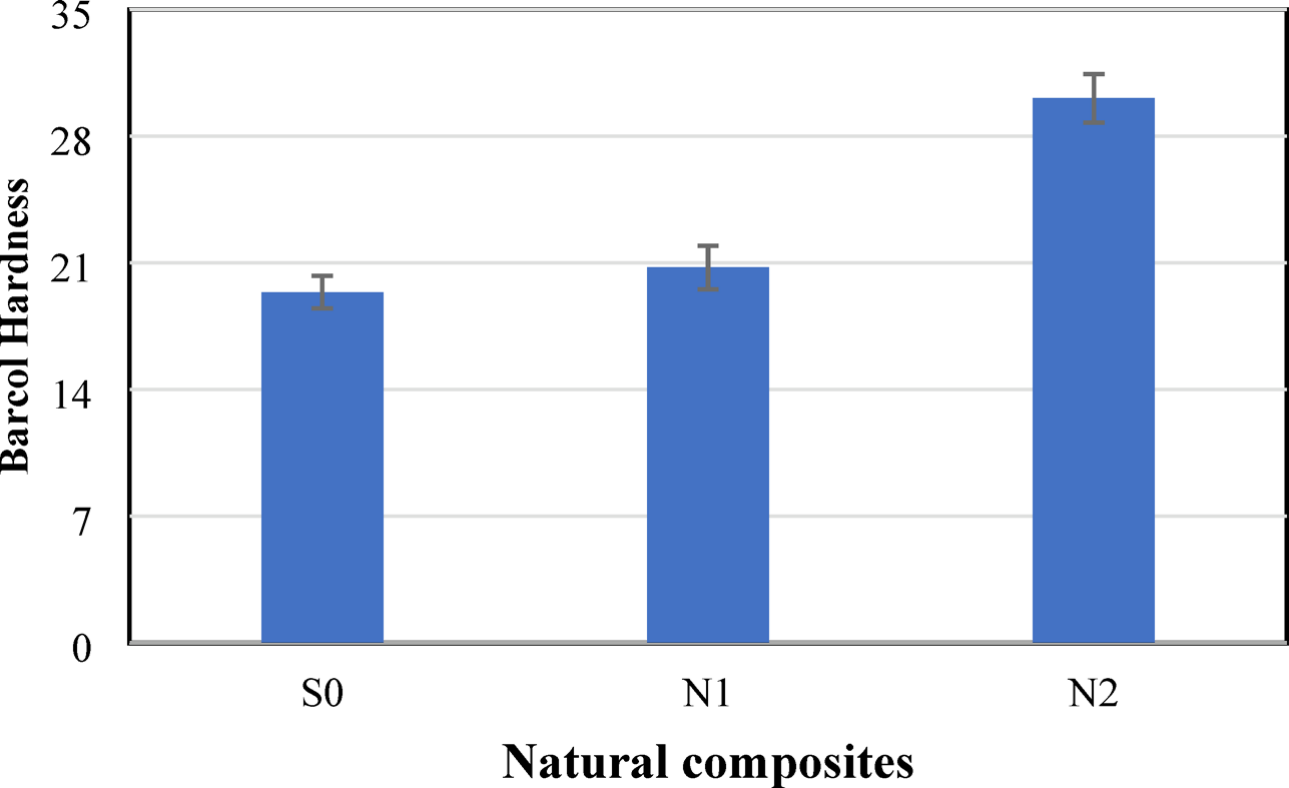
Hardness of GNPs wood epoxy and pure wood epoxy composites.

As illustrated in Fig. 31, the hardness of the wood-epoxy composite exhibited a marginal improvement at a loading of 0.125 wt% graphene nanoplatelets (GNPs). Nevertheless, raising the GNP concentration to 0.25 wt% resulted in a substantial 55% enhancement in hardness compared to the control specimen. The enhancement can be explained that GNPs fill the micro-voids and interstitial spaces within the wood-epoxy structure. This creates a more compact and homogenous material that is less susceptible to penetration by an indenter. Similar findings also obtained by^27,31,59^.

**Fig. 32:**
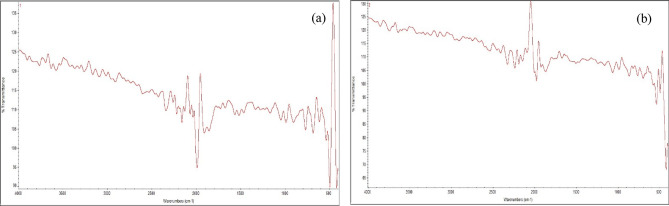
FTIR for wood/epoxy specimens (**a**) 0.125 wt% graphene nanoplatelets and (**b**) 0.25 wt% graphene nanoplatelets.

The FTIR spectra of the wood/epoxy composites with 0.125 wt% and 0.25 wt% graphene nanoplatelets are shown in Fig. 32a, b, respectively. Similar absorption bands are seen in both spectra, suggesting that the basic chemical structure of the wood/epoxy composite was unaffected by the inclusion of GNPs. Slight differences in band intensity, however, point to interactions between the epoxy matrix and the GNPs. In line with the enhanced tensile and flexural qualities noted in the mechanical testing, the 0.125 wt% GNPs composite showed more advantageous spectrum features. On the other hand, only slight spectrum changes occurred when the GNPs content was increased to 0.25 wt%. This is consistent with the limited improvement in mechanical performance, which is probably caused by the commencement of GNPs agglomeration at the higher loading.

**Fig. 33:**
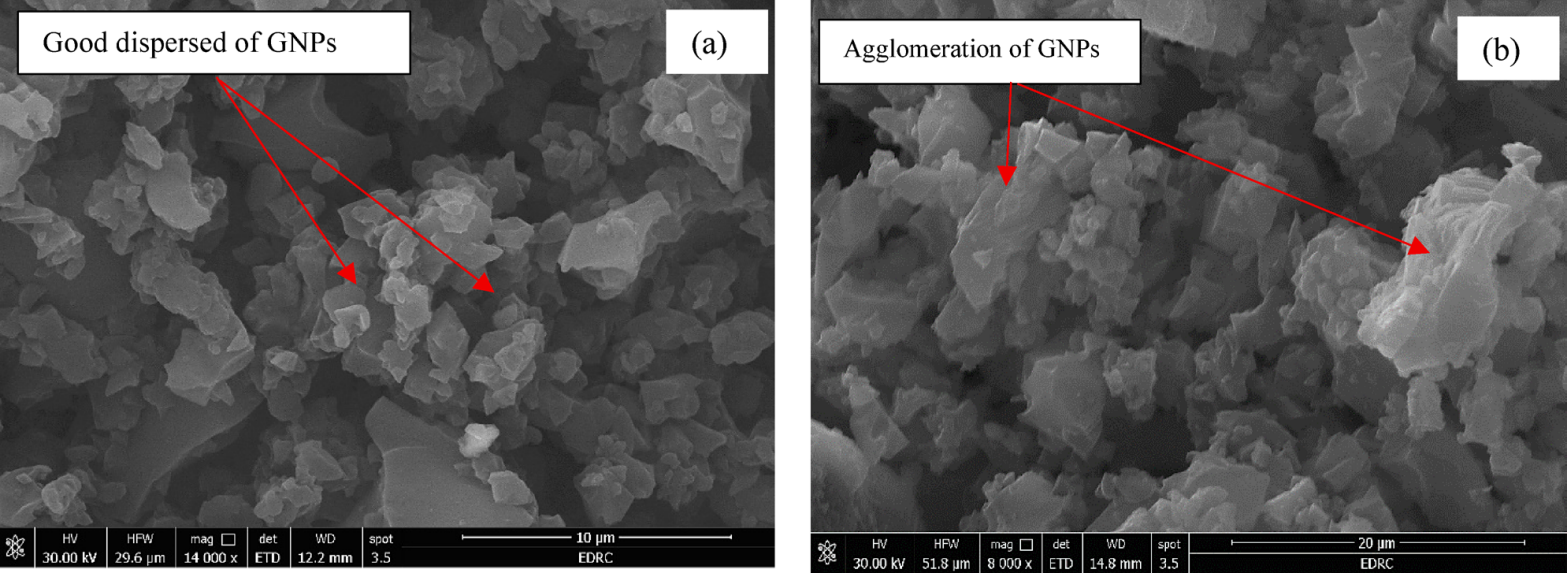
SEM images demonstrating the dispersion of (**a**) N1 and (**b**) N2 specimens.

As shown in Figs. 33a SEM micrographs revealed that the incorporation of 0.125 wt% graphene platelets into the untreated wood-epoxy composites resulted in a highly uniform nanoparticle dispersion. This homogeneous morphology facilitated effective stress distribution throughout the matrix, thereby enhancing the overall mechanical properties. Conversely, increasing the nanomaterial loading to 0.25 wt% GPNs induced significant nanoparticle agglomeration as shown in Fig. 33b These localized clusters acted as stress concentrators, disrupting uniform load transfer and consequently degrading the mechanical performance of the composite bulk system. Also the emergence of an extensive fiber pull-out fracture surface at the higher 0.25 wt% GPNs loading can be attributed to the detrimental effects of nanoparticle agglomeration on the interfacial zone. The formation of GPNs clusters increases the local viscosity of the epoxy resin during processing, which severely restricts resin infiltration and wetting of the wood fibers. Furthermore, these agglomerates drastically reduce the effective surface area available for interfacial interaction, creating microstructural voids and weak boundaries at the fiber-matrix interface. Consequently, the interfacial shear strength is significantly degraded, causing the crack propagation path to divert along the poorly bonded interface and resulting in fiber debonding and pull-out rather than fiber breakage^54^.

As shown in Fig. 33, SEM micrographs illustrate a clear transition in filler morphology between the two GNPs contents, confirming the dispersion limit of the epoxy matrix. In Fig. 33a, representing the lower loading content of 0.125 wt% GNPs, the fillers are characterized as well-dispersed GNPs. At this optimal content, the individual GNPs remain separated and evenly distributed throughout the microstructure. This good distribution maximizes the effective surface area available for interfacial interaction, facilitating efficient stress transfer and activating energy dissipating mechanisms.

Conversely, Fig. 33 (b) shows the morphology at the higher loading of 0.25 wt.% GNPs, which is dominated by the agglomeration of GNPs. Increasing the GNPs content allows attractive van der Waals forces to overcome matrix shearing, causing the GNPs to cluster into dense, multi-layered aggregates. These macroscopic agglomerates not only reduce the effective reinforcement surface area but also act as internal structural defects. They entrap air, block local resin infiltration, and create localized stress concentration sites, directly explaining the subsequent degradation in both tensile and flexural strengths observed at higher loading contents.

#### Thermogravimetric analysis

Figure 23. shows the Thermogravimetric Analysis (TGA) for GNPs wood epoxy composites. The TGA graph compares the heat resistance of the untreated wood composite, a low-graphene content sample, and a high-graphene content sampleThe figures indicated that adding graphene nanoplatelets to the wood composite results in a concentration-dependent thermal response. A low concentration of 0.125% GNPs reduces thermal stability because it accelerates internal heat transfer, while a higher concentration of 0.25% GNPs significantly improves stability by creating a physical shield that delays major burning. Ultimately, the higher loading slows down active decomposition but leads to a more complete burn-off at extreme temperatures^60^. The nanocomposite, N2 exhibited a slight shift of the main degradation region toward elevated temperatures compared with the untreated composite S0, indicating improved thermal stability due to the presence of GNPs. However, the residual weight at the end of the test was slightly lower than that of S0, suggesting that the addition of GNPs mainly delayed the degradation process rather than increasing the final burn residue. Among the investigated formulations, N2 exhibited the highest thermal stability, confirming that the incorporation of graphene nanoplatelets effectively delays the thermal degradation of the wood/epoxy composites. However, Fig. 24. Shows the Derivative Thermogravimetric analysis for GNPs wood epoxy composites. The addition of graphene nanoplatelets shifted the maximum degradation peak toward higher temperatures compared to the unfilled composite, indicating enhanced thermal stability. The N1 composite exhibited an intermediate degradation temperature between S0 and N2, demonstrating that even lower GNPs content effectively delayed the degradation process. This improvement is attributed to the barrier effect of the graphene nanoplatelets, which hinder heat transfer and the diffusion of volatile degradation products. Among the investigated composites, N2 exhibited the highest degradation temperature, confirming that the higher GNP content provided the greatest improvement in thermal resistance.

**Fig. 34:**
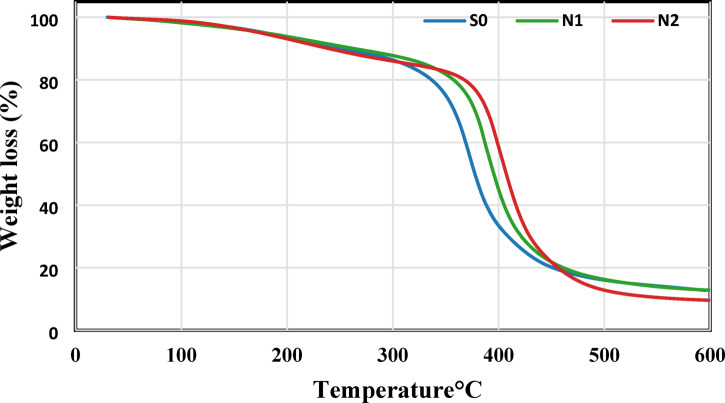
Thermogravimetric Analysis (TGA) for GNPs wood epoxy composites.

**Fig. 35:**
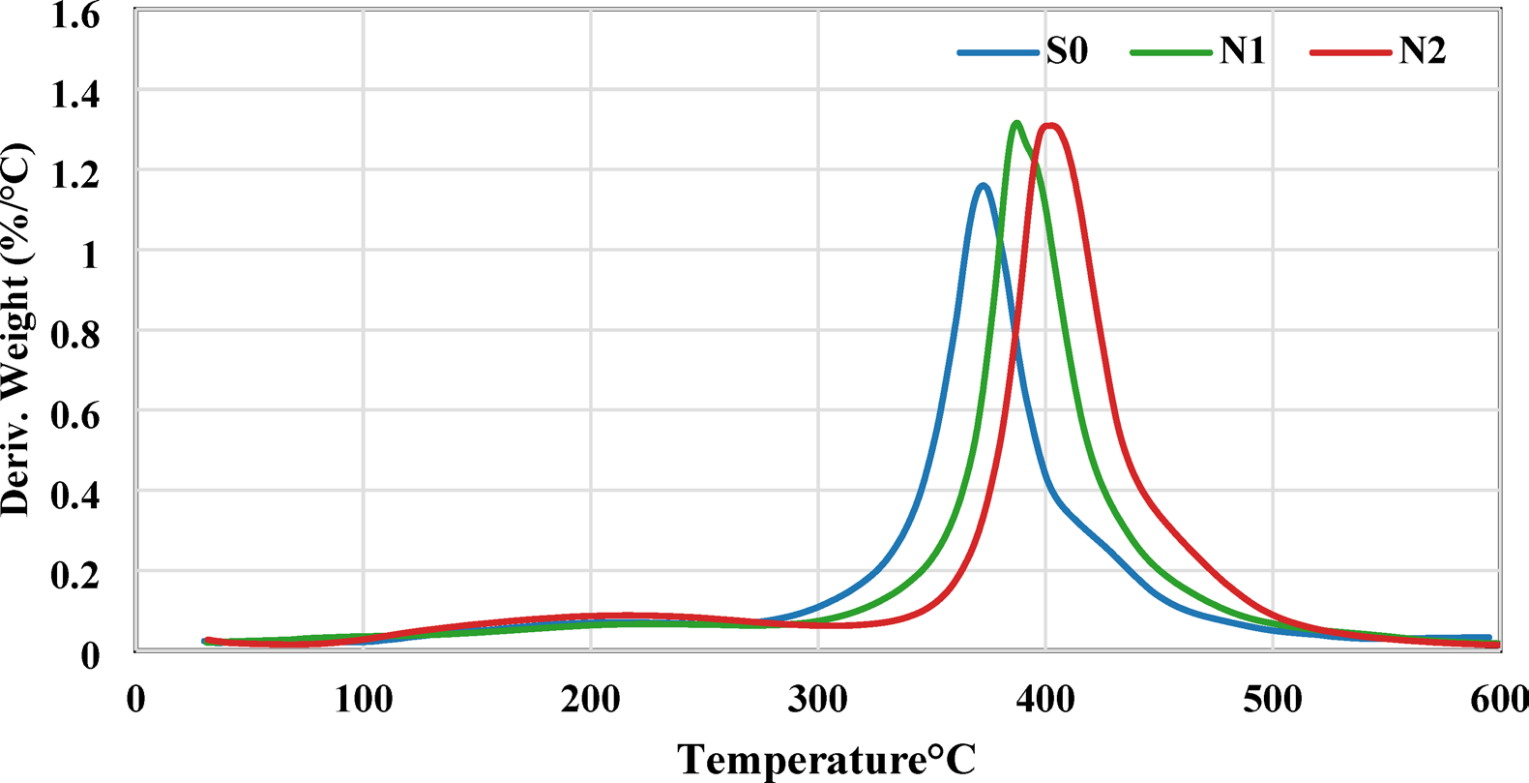
Derivative Thermogravimetric analysis for GNPs wood epoxy composites.

## Conclusions

Graphene nanoplatelet and alkaline surface treatment of wood fiber were used to create different types of wood-epoxy composites. Two types of wood short fibers were used, Swedish and pitch pine wood. Wood fibers were treated with two concentrations 5% and 10% NaOH, however, GNPs was added to the epoxy matrix at weight percentages of 0.125 and 0.25. The results revealed that 5% of the wood composites exhibit the best tensile, flexural and impact performance due to an optimal balance between surface modification and fiber integrity while higher alkali concentration (10%) negatively affects the mechanical performance as compared to untreated wood/epoxy composites. Composites with 0.125% GNPs have the highest tensile strength, flexural strength, and flexural strain as compared to unfilled wood/epoxy composite. However, increasing the nanofiller loading to 0.25 wt.% resulted in a progressive improvement in hardness and impact energy absorption of the wood composites. Overall, an efficient method for creating sustainable wood-epoxy composites with improved mechanical performance is the addition of 5% alkali-treated wood fibers and 0.125% GNP content. These composites are promising for furniture, construction panels, automotive interior parts, decorative structures, and other structural products where increased strength, stiffness, hardness and impact resistance.

## Data Availability

The datasets used during the current study are available from the corresponding author on reasonable request.
